# Supplementary material for: Exploring joint decision-making and family dynamics to identify barriers and enablers for early adolescent medical circumcision (EAMC) uptake in Zambia for HIV prevention: An innovative methodology
Source: PLoS One. 2025 Apr 29;20(4):e0319472. doi: 10.1371/journal.pone.0319472 (PMC12040159; doi:10.1371/journal.pone.0319472)
Supplement: S1 File — (PPTX) [file pone.0319472.s001.pptx]

## Slide 1
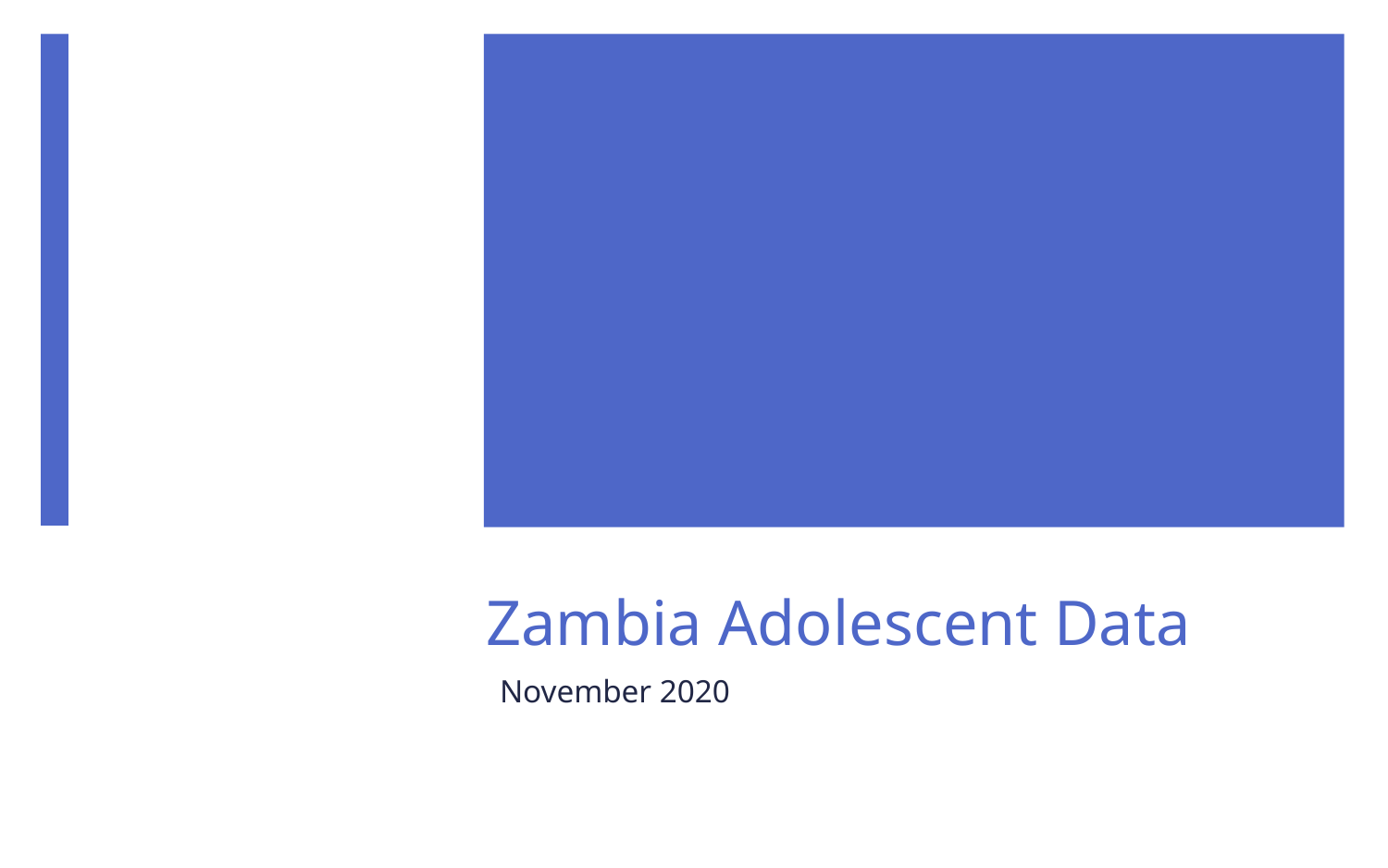

# Zambia Adolescent Data
November 2020

## Slide 2
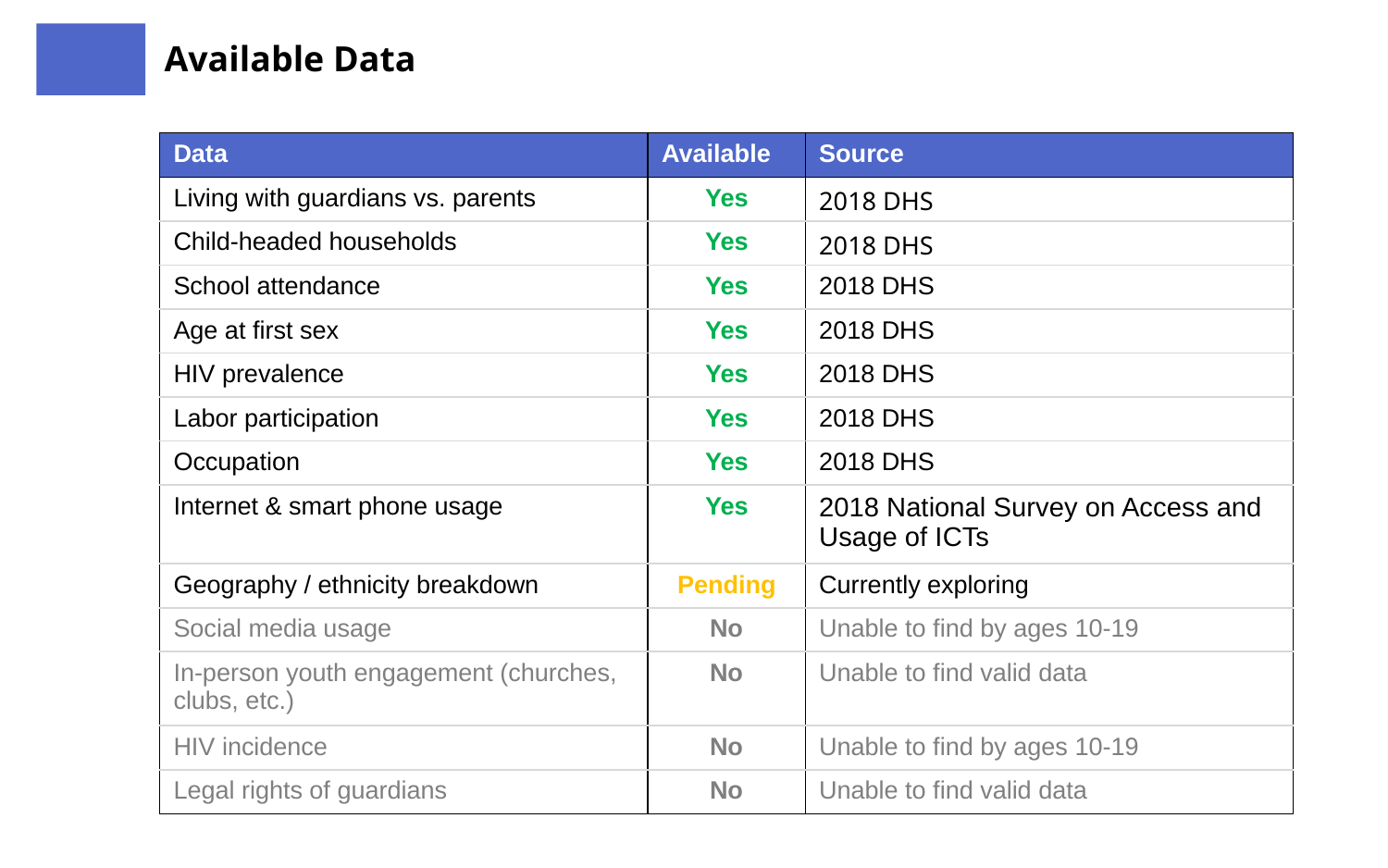

Available Data
| Data | Available | Source |
| --- | --- | --- |
| Living with guardians vs. parents | Yes | 2018 DHS |
| Child-headed households | Yes | 2018 DHS |
| School attendance | Yes | 2018 DHS |
| Age at first sex | Yes | 2018 DHS |
| HIV prevalence | Yes | 2018 DHS |
| Labor participation | Yes | 2018 DHS |
| Occupation | Yes | 2018 DHS |
| Internet & smart phone usage | Yes | 2018 National Survey on Access and Usage of ICTs |
| Geography / ethnicity breakdown | Pending | Currently exploring |
| Social media usage | No | Unable to find by ages 10-19 |
| In-person youth engagement (churches, clubs, etc.) | No | Unable to find valid data |
| HIV incidence | No | Unable to find by ages 10-19 |
| Legal rights of guardians | No | Unable to find valid data |

## Slide 3
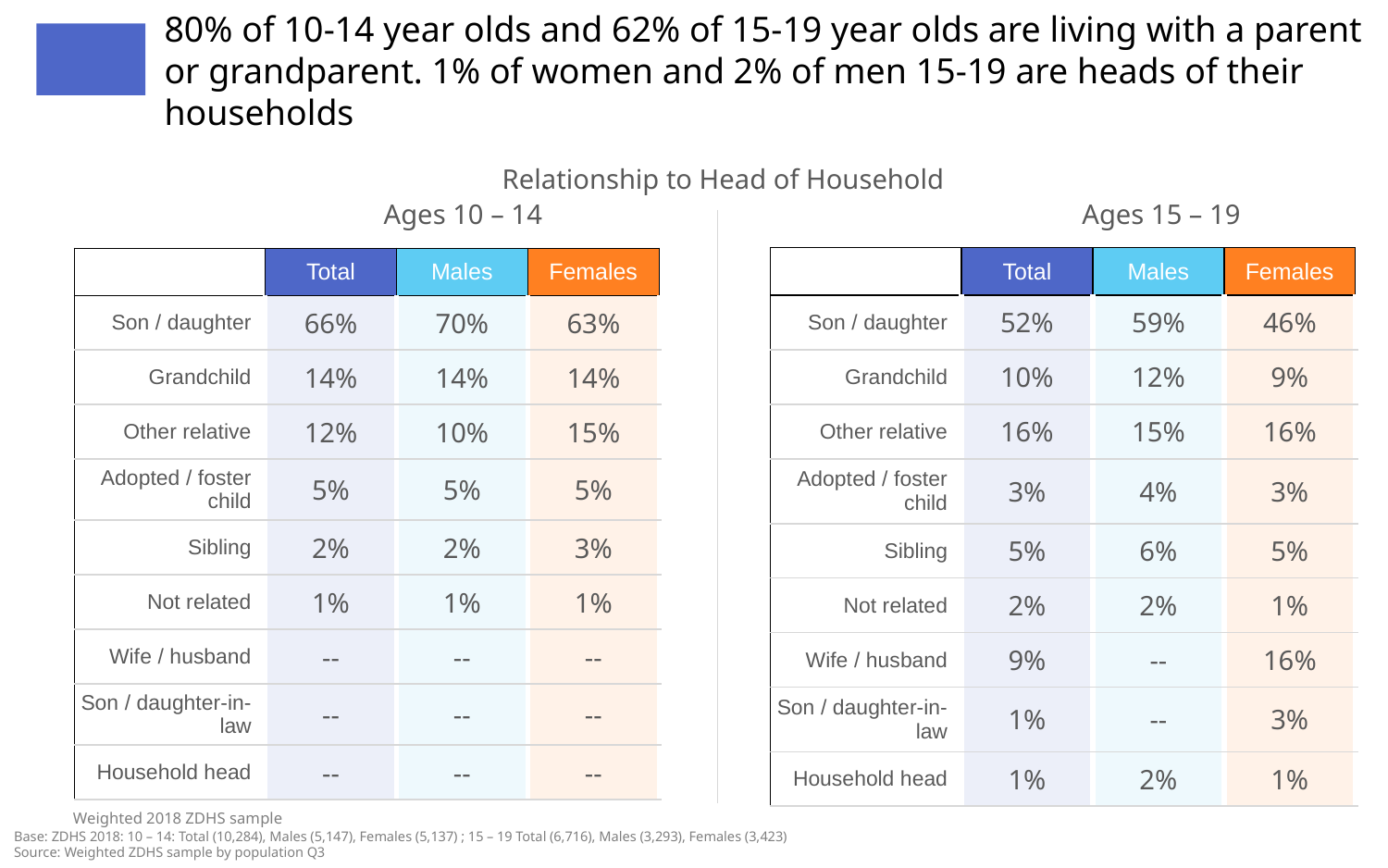

80% of 10-14 year olds and 62% of 15-19 year olds are living with a parent or grandparent. 1% of women and 2% of men 15-19 are heads of their households
Relationship to Head of Household
Ages 15 – 19
Ages 10 – 14
| | Total | Males | Females |
| --- | --- | --- | --- |
| Son / daughter | 52% | 59% | 46% |
| Grandchild | 10% | 12% | 9% |
| Other relative | 16% | 15% | 16% |
| Adopted / foster child | 3% | 4% | 3% |
| Sibling | 5% | 6% | 5% |
| Not related | 2% | 2% | 1% |
| Wife / husband | 9% | -- | 16% |
| Son / daughter-in-law | 1% | -- | 3% |
| Household head | 1% | 2% | 1% |
| | Total | Males | Females |
| --- | --- | --- | --- |
| Son / daughter | 66% | 70% | 63% |
| Grandchild | 14% | 14% | 14% |
| Other relative | 12% | 10% | 15% |
| Adopted / foster child | 5% | 5% | 5% |
| Sibling | 2% | 2% | 3% |
| Not related | 1% | 1% | 1% |
| Wife / husband | -- | -- | -- |
| Son / daughter-in-law | -- | -- | -- |
| Household head | -- | -- | -- |
Weighted 2018 ZDHS sample
Base: ZDHS 2018: 10 – 14: Total (10,284), Males (5,147), Females (5,137) ; 15 – 19 Total (6,716), Males (3,293), Females (3,423)
Source: Weighted ZDHS sample by population Q3

## Slide 4
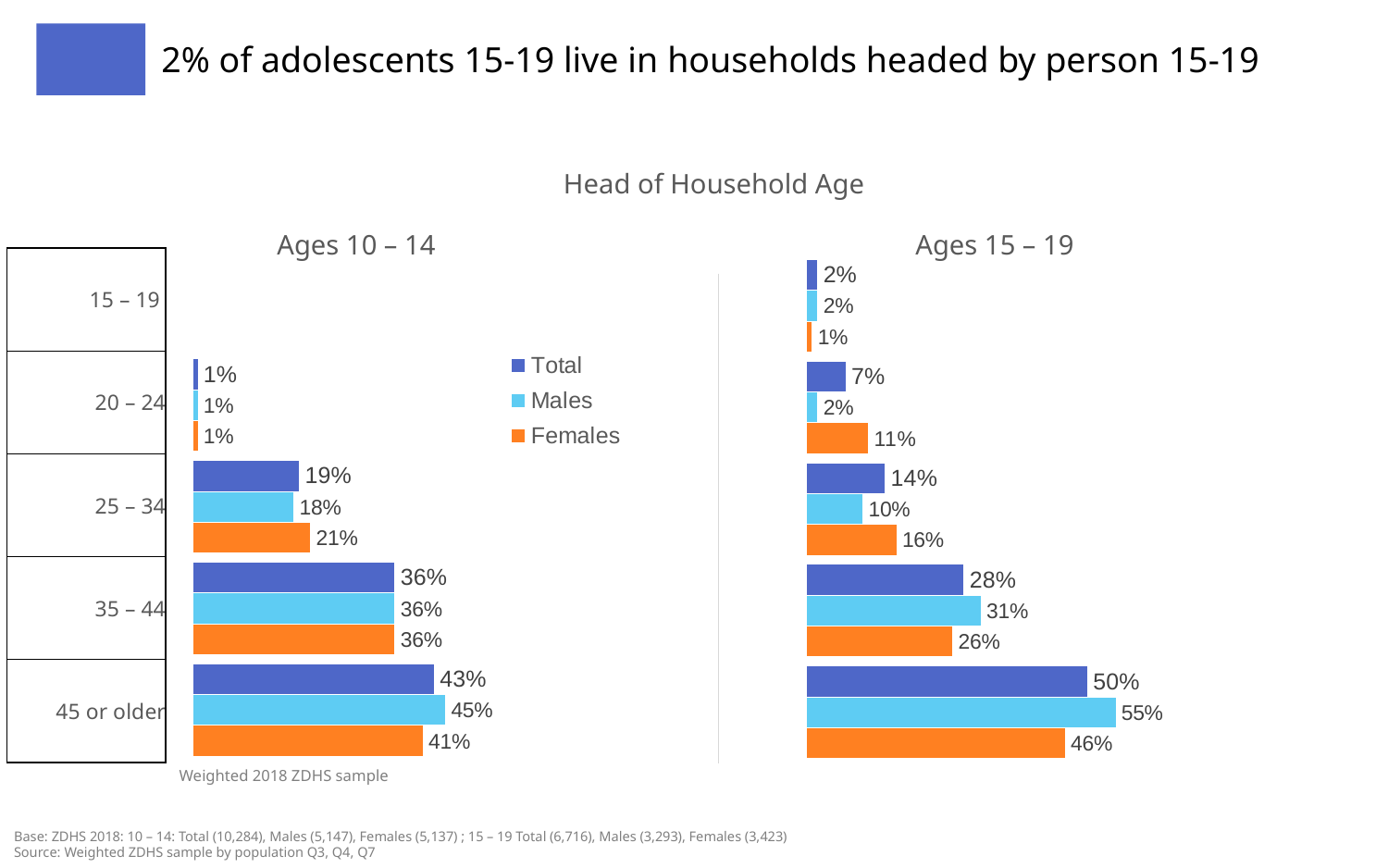

2% of adolescents 15-19 live in households headed by person 15-19
Head of Household Age
Ages 15 – 19
Ages 10 – 14
### Chart
| Category | Females | Males | Total |
|---|---|---|---|
| 45 and older | 0.41 | 0.45 | 0.43 |
| 35 to 44 | 0.36 | 0.36 | 0.36 |
| 25 to 34 | 0.21 | 0.18 | 0.19 |
| 20 to 24 | 0.01 | 0.01 | 0.01 |
| 15 to 19 | None | None | None |
### Chart
| Category | Females | Males | Total |
|---|---|---|---|
| 45 and older | 0.46 | 0.55 | 0.5 |
| 35 to 44 | 0.26 | 0.31 | 0.28 |
| 25 to 34 | 0.16 | 0.1 | 0.14 |
| 20 to 24 | 0.11 | 0.02 | 0.07 |
| 15 to 19 | 0.01 | 0.02 | 0.02 || 15 – 19 |
| --- |
| 20 – 24 |
| 25 – 34 |
| 35 – 44 |
| 45 or older |
Weighted 2018 ZDHS sample
Base: ZDHS 2018: 10 – 14: Total (10,284), Males (5,147), Females (5,137) ; 15 – 19 Total (6,716), Males (3,293), Females (3,423)
Source: Weighted ZDHS sample by population Q3, Q4, Q7

## Slide 5
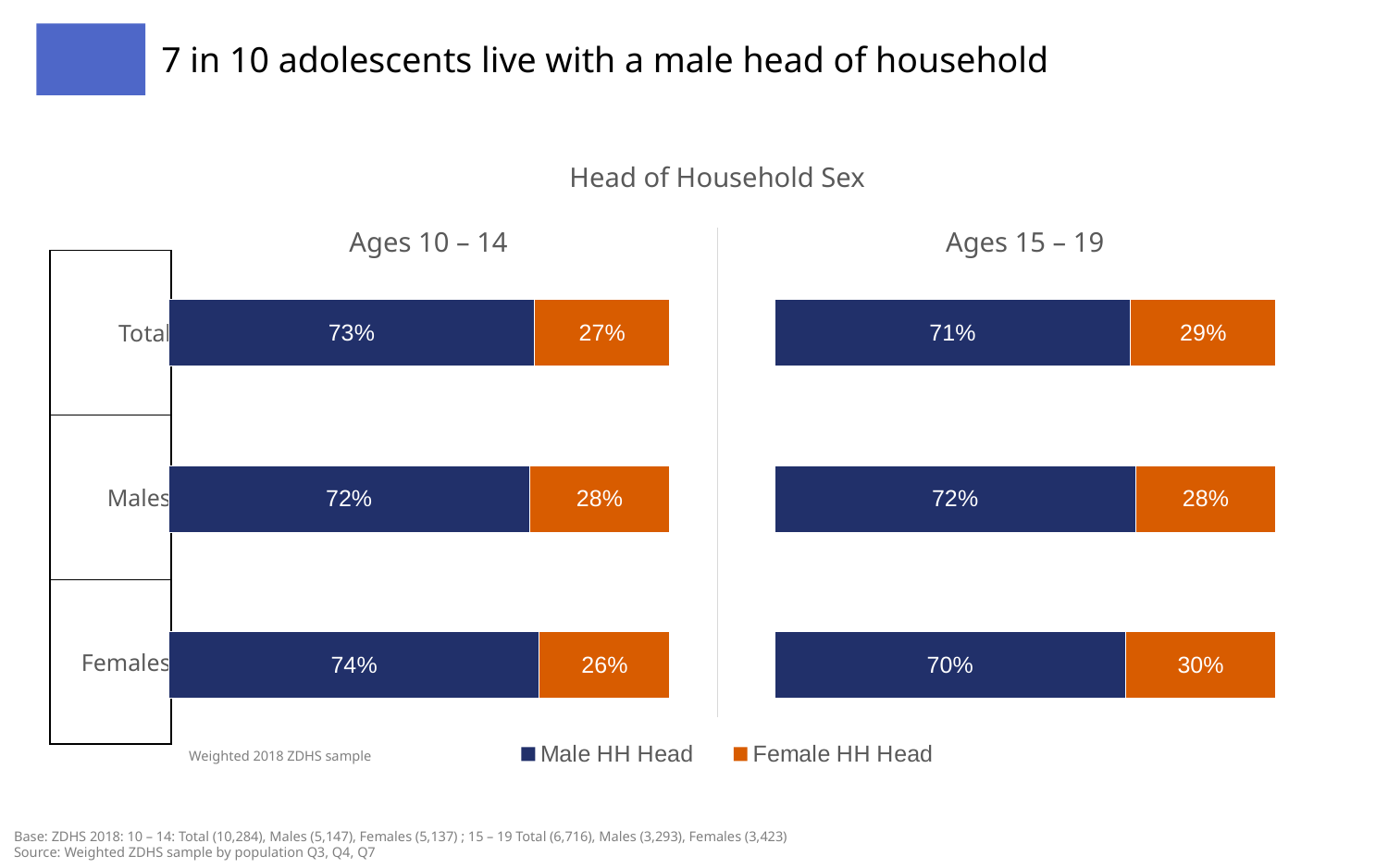

7 in 10 adolescents live with a male head of household
Head of Household Sex
Ages 15 – 19
Ages 10 – 14
### Chart
| Category | Series 1 | Series 2 |
|---|---|---|
| Category 1 | 0.74 | 0.26 |
| Category 2 | 0.72 | 0.28 |
| Category 3 | 0.73 | 0.27 |
### Chart
| Category | Series 1 | Series 2 |
|---|---|---|
| Category 1 | 0.7 | 0.3 |
| Category 2 | 0.72 | 0.28 |
| Category 3 | 0.71 | 0.29 || Total |
| --- |
| Males |
| Females |
### Chart
| Category | Column1 |
|---|---|
| Male HH Head | None |
| Female HH Head | None |Weighted 2018 ZDHS sample
Base: ZDHS 2018: 10 – 14: Total (10,284), Males (5,147), Females (5,137) ; 15 – 19 Total (6,716), Males (3,293), Females (3,423)
Source: Weighted ZDHS sample by population Q3, Q4, Q7

## Slide 6
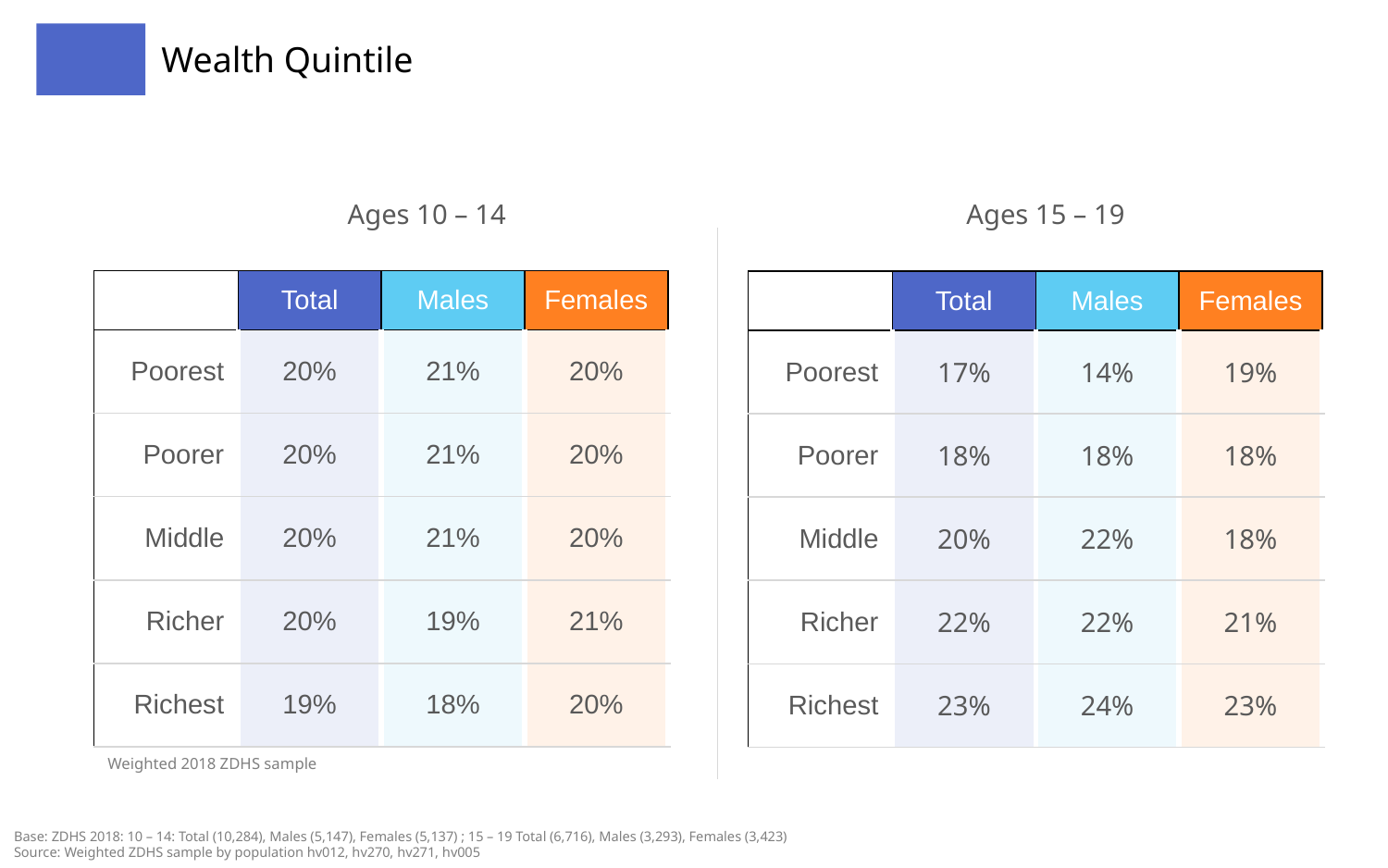

Wealth Quintile
Ages 15 – 19
Ages 10 – 14
| | Total | Males | Females |
| --- | --- | --- | --- |
| Poorest | 20% | 21% | 20% |
| Poorer | 20% | 21% | 20% |
| Middle | 20% | 21% | 20% |
| Richer | 20% | 19% | 21% |
| Richest | 19% | 18% | 20% |
| | Total | Males | Females |
| --- | --- | --- | --- |
| Poorest | 17% | 14% | 19% |
| Poorer | 18% | 18% | 18% |
| Middle | 20% | 22% | 18% |
| Richer | 22% | 22% | 21% |
| Richest | 23% | 24% | 23% |
Weighted 2018 ZDHS sample
Base: ZDHS 2018: 10 – 14: Total (10,284), Males (5,147), Females (5,137) ; 15 – 19 Total (6,716), Males (3,293), Females (3,423)
Source: Weighted ZDHS sample by population hv012, hv270, hv271, hv005

## Slide 7
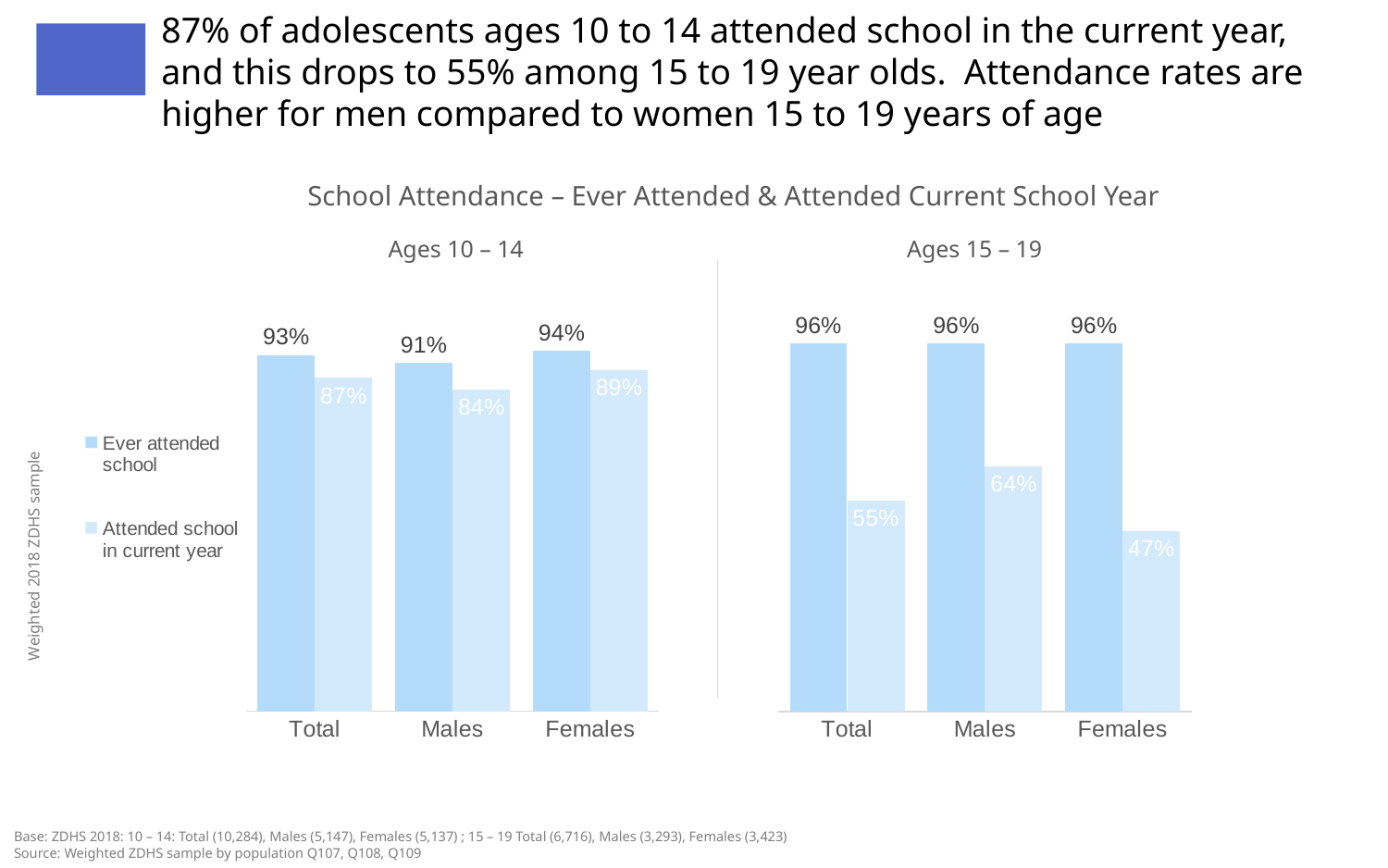

87% of adolescents ages 10 to 14 attended school in the current year, and this drops to 55% among 15 to 19 year olds. Attendance rates are higher for men compared to women 15 to 19 years of age
School Attendance – Ever Attended & Attended Current School Year
Ages 15 – 19
Ages 10 – 14
### Chart
| Category | Ever attended school | Attended school in current year |
|---|---|---|
| Total | 0.93 | 0.87 |
| Males | 0.91 | 0.84 |
| Females | 0.94 | 0.89 |
### Chart
| Category | Ever attended school | Attended school in current year |
|---|---|---|
| Total | 0.96 | 0.55 |
| Males | 0.96 | 0.64 |
| Females | 0.96 | 0.47 |
### Chart
| Category | Ever attended school | Attended school in current year |
|---|---|---|
| Total | None | None |
| Males | None | None |
| Females | None | None |Weighted 2018 ZDHS sample
Base: ZDHS 2018: 10 – 14: Total (10,284), Males (5,147), Females (5,137) ; 15 – 19 Total (6,716), Males (3,293), Females (3,423)
Source: Weighted ZDHS sample by population Q107, Q108, Q109

## Slide 8
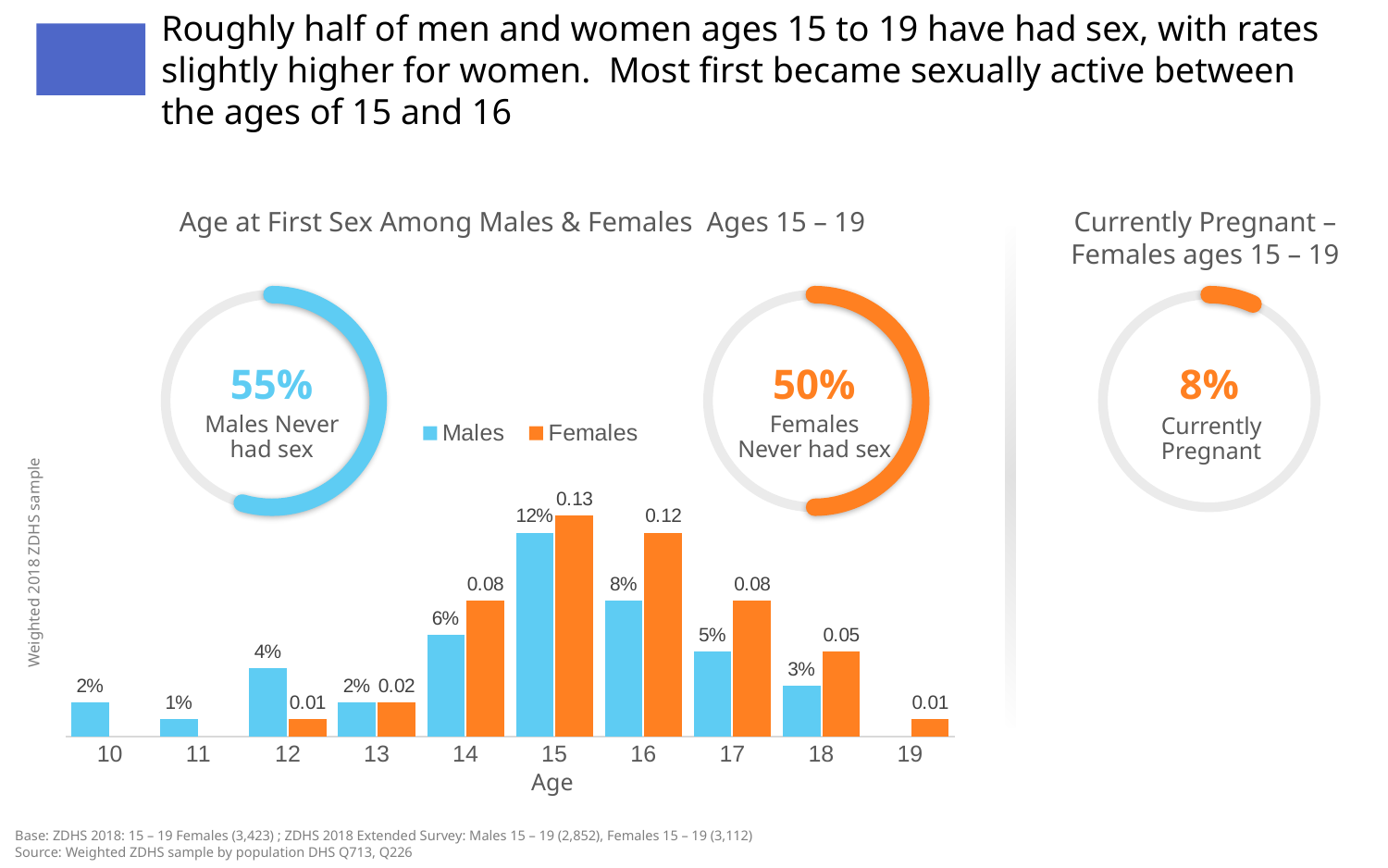

Roughly half of men and women ages 15 to 19 have had sex, with rates slightly higher for women. Most first became sexually active between the ages of 15 and 16
Age at First Sex Among Males & Females Ages 15 – 19
Currently Pregnant – Females ages 15 – 19
55%
Males Never had sex
50%
Females Never had sex
8%
Currently Pregnant
### Chart
| Category | Males | Females |
|---|---|---|
| 10 | 0.02 | None |
| 11 | 0.01 | None |
| 12 | 0.04 | 0.01 |
| 13 | 0.02 | 0.02 |
| 14 | 0.06 | 0.08 |
| 15 | 0.12 | 0.13 |
| 16 | 0.08 | 0.12 |
| 17 | 0.05 | 0.08 |
| 18 | 0.03 | 0.05 |
| 19 | None | 0.01 |Weighted 2018 ZDHS sample
Age
Base: ZDHS 2018: 15 – 19 Females (3,423) ; ZDHS 2018 Extended Survey: Males 15 – 19 (2,852), Females 15 – 19 (3,112)
Source: Weighted ZDHS sample by population DHS Q713, Q226

## Slide 9
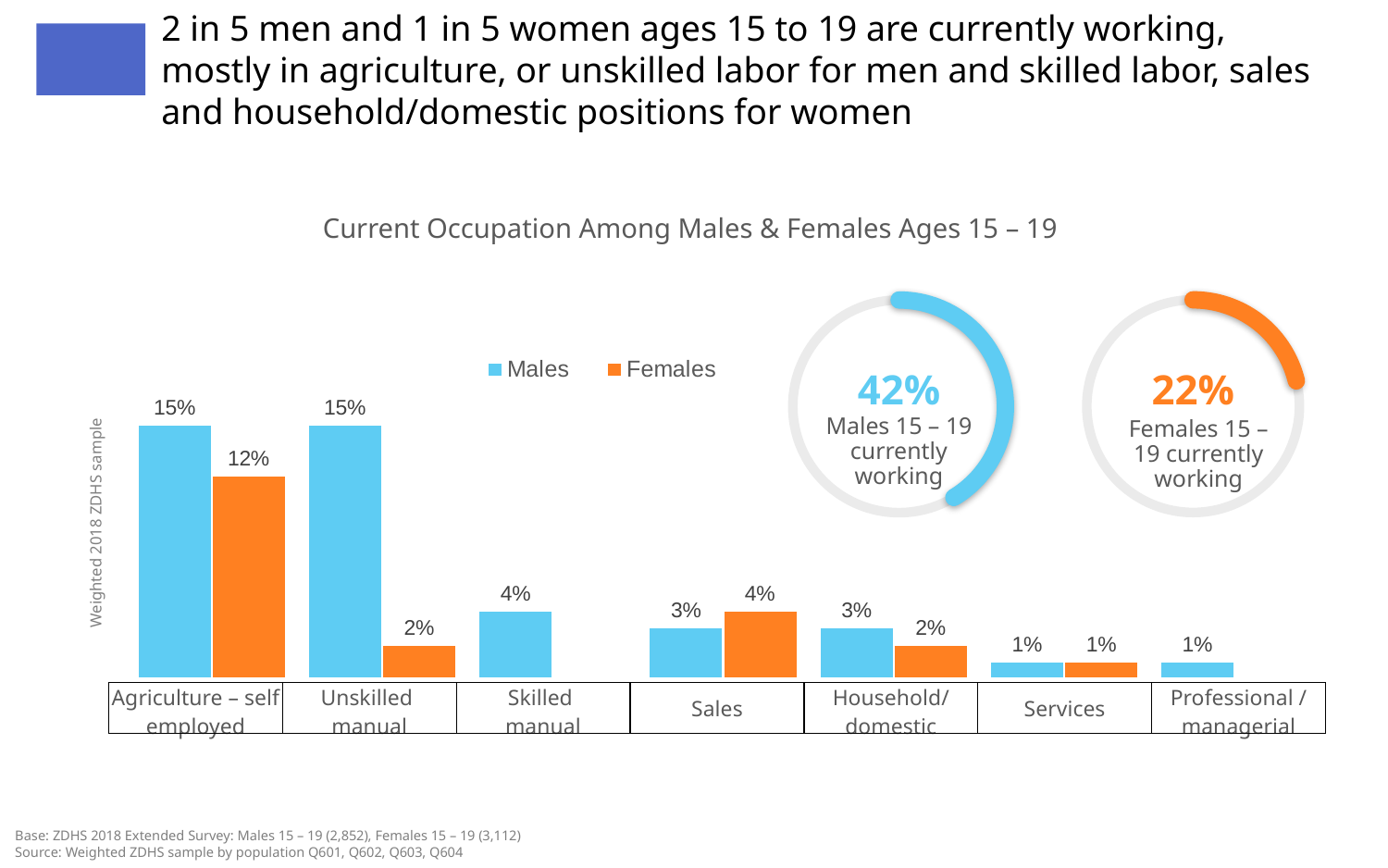

2 in 5 men and 1 in 5 women ages 15 to 19 are currently working, mostly in agriculture, or unskilled labor for men and skilled labor, sales and household/domestic positions for women
Current Occupation Among Males & Females Ages 15 – 19
22%
Females 15 – 19 currently working
42%
Males 15 – 19 currently working
### Chart
| Category | Males | Females |
|---|---|---|
| Agriculture - self employed | 0.15 | 0.12 |
| Unskilled Manual | 0.15 | 0.02 |
| Skilled manual | 0.04 | None |
| Sales | 0.03 | 0.04 |
| Household & domestic | 0.03 | 0.02 |
| Services | 0.01 | 0.01 |
| Professional / managerial | 0.01 | None |Weighted 2018 ZDHS sample
| Agriculture – self employed | Unskilled manual | Skilled manual | Sales | Household/ domestic | Services | Professional / managerial |
| --- | --- | --- | --- | --- | --- | --- |
Base: ZDHS 2018 Extended Survey: Males 15 – 19 (2,852), Females 15 – 19 (3,112)
Source: Weighted ZDHS sample by population Q601, Q602, Q603, Q604

## Slide 10
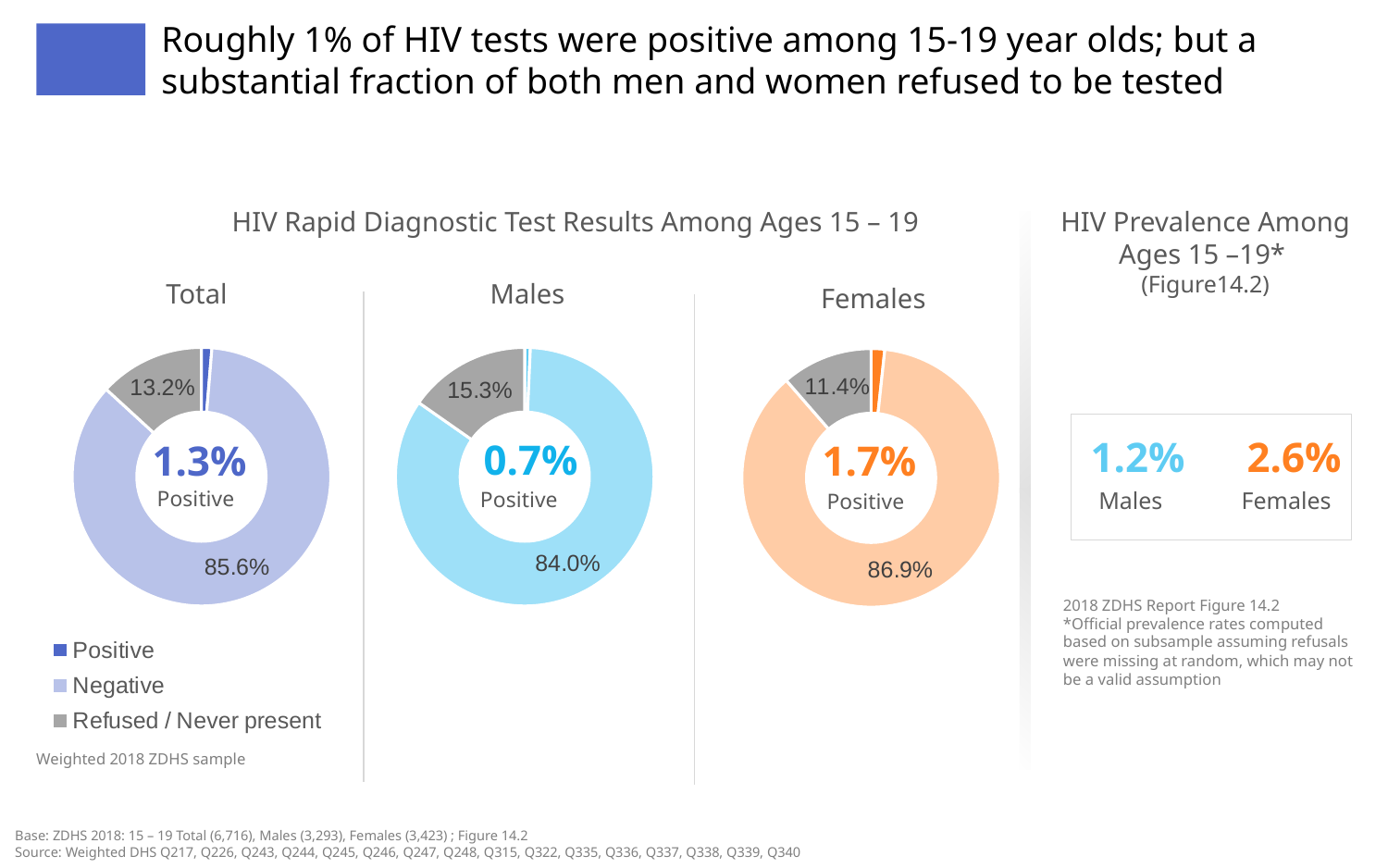

Roughly 1% of HIV tests were positive among 15-19 year olds; but a substantial fraction of both men and women refused to be tested
HIV Rapid Diagnostic Test Results Among Ages 15 – 19
HIV Prevalence Among Ages 15 –19*
(Figure14.2)
Total
Males
Females
### Chart
| Category | Sales |
|---|---|
| Positive | 0.013 |
| Negative | 0.856 |
| Refused / Never present | 0.132 |
### Chart
| Category | Sales |
|---|---|
| Positive | 0.007 |
| Negative | 0.84 |
| Refused / Never present | 0.153 |
### Chart
| Category | Sales |
|---|---|
| Positive | 0.017 |
| Negative | 0.869 |
| Refused / Never present | 0.114 |
1.2%
2.6%
0.7%
1.3%
1.7%
Positive
Positive
Males
Females
Positive
2018 ZDHS Report Figure 14.2
*Official prevalence rates computed based on subsample assuming refusals were missing at random, which may not be a valid assumption
Weighted 2018 ZDHS sample
Base: ZDHS 2018: 15 – 19 Total (6,716), Males (3,293), Females (3,423) ; Figure 14.2
Source: Weighted DHS Q217, Q226, Q243, Q244, Q245, Q246, Q247, Q248, Q315, Q322, Q335, Q336, Q337, Q338, Q339, Q340

## Slide 11
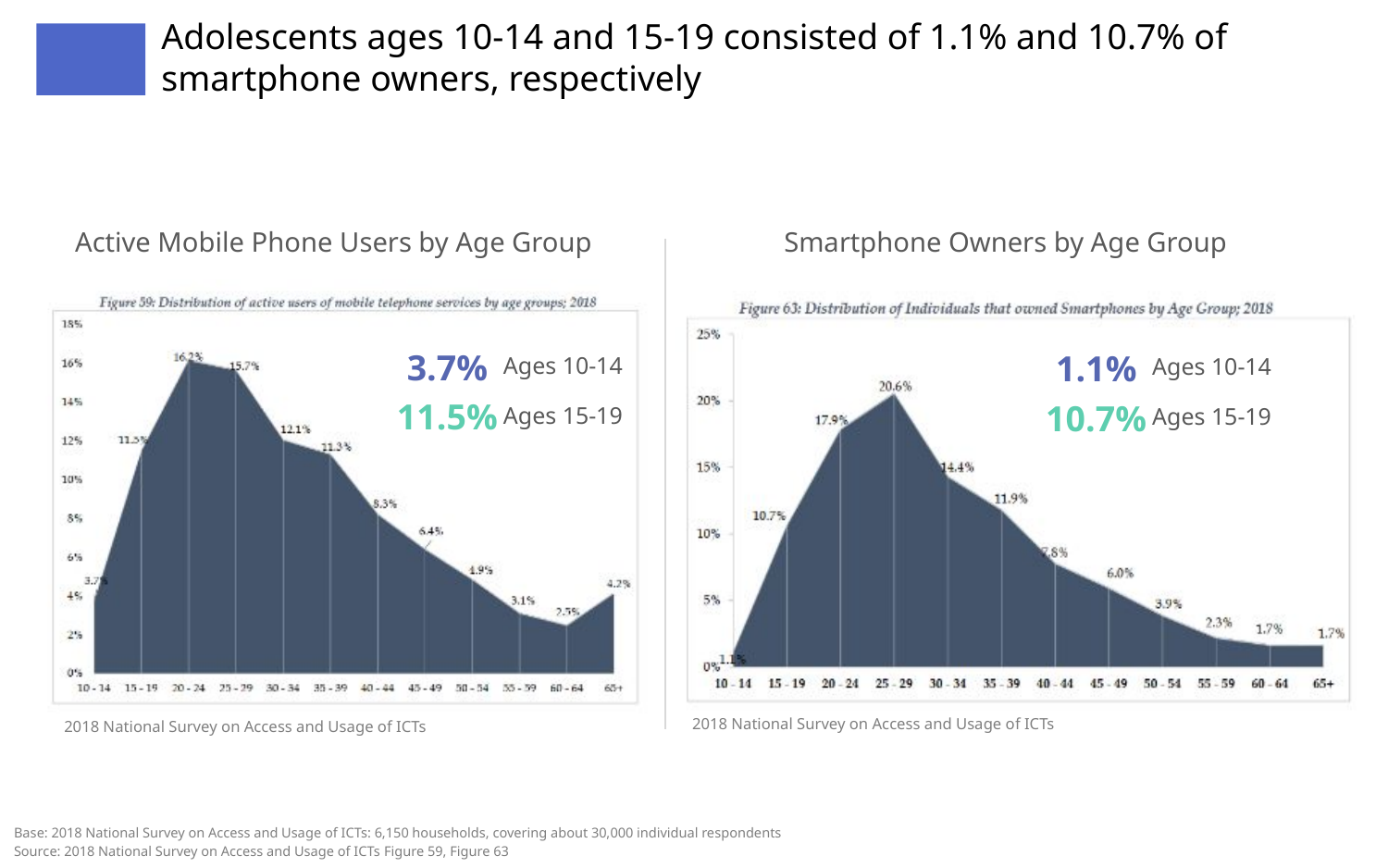

Adolescents ages 10-14 and 15-19 consisted of 1.1% and 10.7% of smartphone owners, respectively
Active Mobile Phone Users by Age Group
Smartphone Owners by Age Group
3.7%
1.1%
Ages 10-14
Ages 10-14
11.5%
10.7%
Ages 15-19
Ages 15-19
2018 National Survey on Access and Usage of ICTs
2018 National Survey on Access and Usage of ICTs
Base: 2018 National Survey on Access and Usage of ICTs: 6,150 households, covering about 30,000 individual respondents
Source: 2018 National Survey on Access and Usage of ICTs Figure 59, Figure 63

## Slide 12
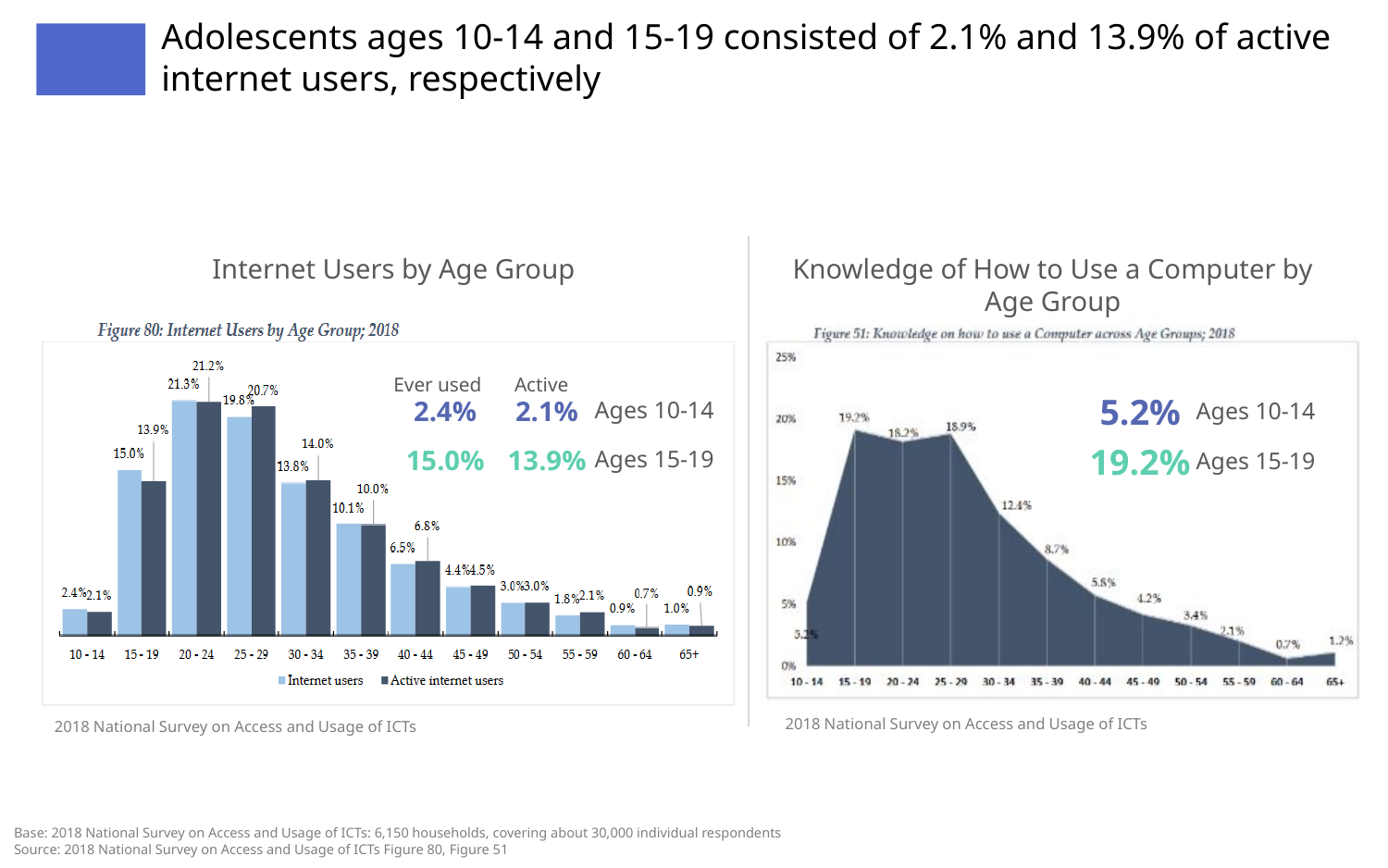

Adolescents ages 10-14 and 15-19 consisted of 2.1% and 13.9% of active internet users, respectively
Internet Users by Age Group
Knowledge of How to Use a Computer by Age Group
Ever used
Active
5.2%
2.4%
2.1%
Ages 10-14
Ages 10-14
19.2%
15.0%
13.9%
Ages 15-19
Ages 15-19
2018 National Survey on Access and Usage of ICTs
2018 National Survey on Access and Usage of ICTs
Base: 2018 National Survey on Access and Usage of ICTs: 6,150 households, covering about 30,000 individual respondents
Source: 2018 National Survey on Access and Usage of ICTs Figure 80, Figure 51

## Slide 13
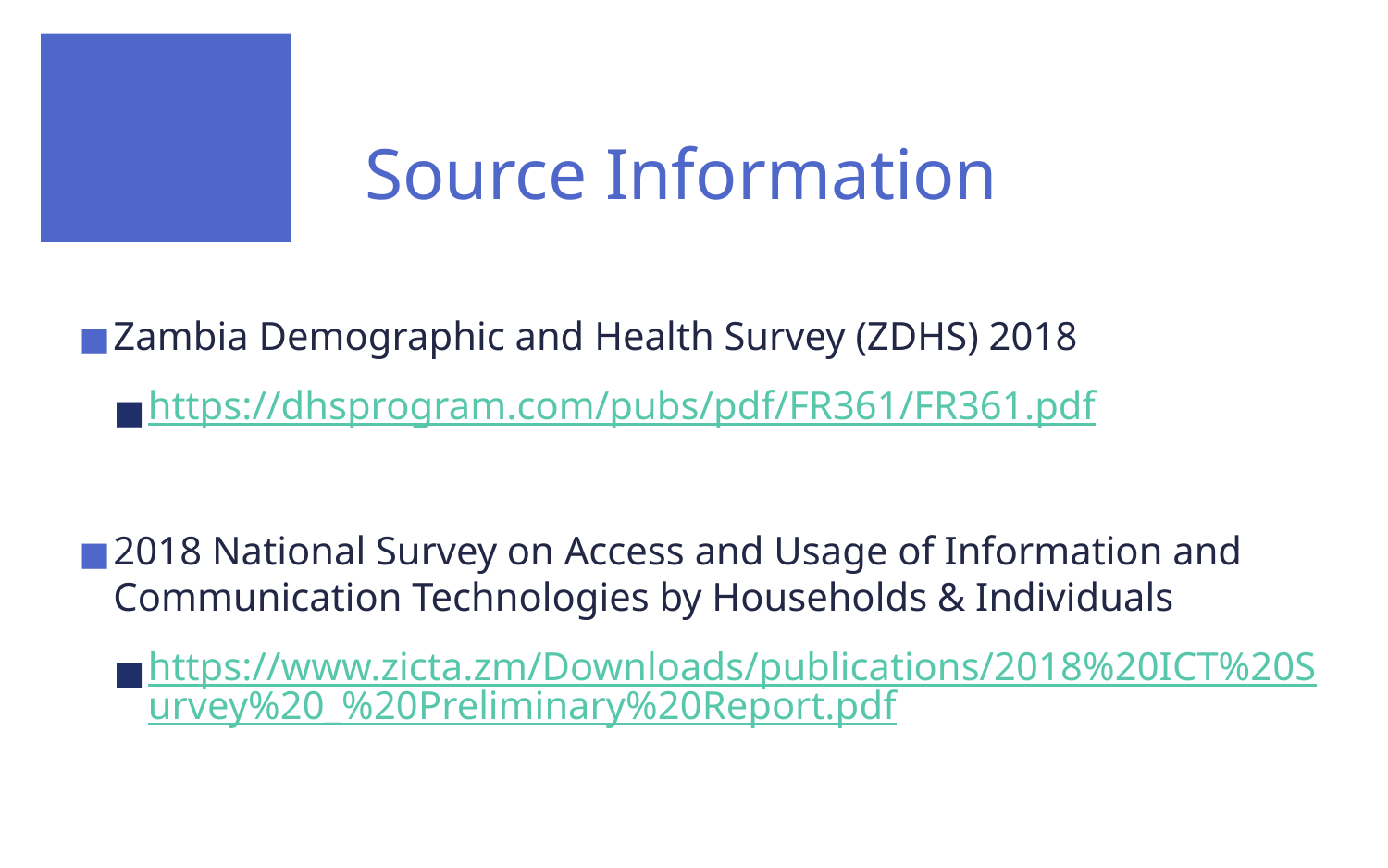

# Source Information
Zambia Demographic and Health Survey (ZDHS) 2018
https://dhsprogram.com/pubs/pdf/FR361/FR361.pdf
2018 National Survey on Access and Usage of Information and Communication Technologies by Households & Individuals
https://www.zicta.zm/Downloads/publications/2018%20ICT%20Survey%20_%20Preliminary%20Report.pdf
