# Supplementary material for: Exploring joint decision-making and family dynamics to identify barriers and enablers for early adolescent medical circumcision (EAMC) uptake in Zambia for HIV prevention: An innovative methodology
Source: PLoS One. 2025 Apr 29;20(4):e0319472. doi: 10.1371/journal.pone.0319472 (PMC12040159; doi:10.1371/journal.pone.0319472)
Supplement: S2 File — (PDF) [file pone.0319472.s002.pdf]

TITLE OF STORY:

A-tarian	Actively Aligning		Not Advocating
A-tative	Anticipating		Advocating
P-missive	young man	father figure	mother figure
indentifier #			

CHAPTER TITLE:

why this title?

BEFORE THE CHANGE

Why did the change happen? What was the motivation?

Reasons **Against**
1.
2.
3.

young man

Reasons **For**
1.
2.
3.

Why I couldn't get them to agree with me

Reasons **Against**
1.
2.
3.

father figure

Reasons **For**
1.
2.
3.

Why I couldn't get them to agree with me

Reasons **Against**
1.
2.
3.

mother figure

Reasons **For**
1.
2.
3.

Why I couldn't get them to agree with me

CHAPTER TITLE:

why this title?

THE CHANGE

How did the change happen? What started it?

Who / What
Channel
Influencer Message
young man
Reaction

My role in this change

Who / What
Channel
Influencer Message
father figure
Reaction

My role in this change

Who / What
Channel
Influencer Message
mother figure
Reaction

My role in this change

CHAPTER TITLE:

why this title?

TODAY

What allowed the family to change? Why?

young man

Driver / Approver

father figure

Driver / Approver

mother figure

Driver / Approver

A-tarian	Actively Aligning	Not Advocating
A-tative	Anticipating	Advocating
P-missive	young man / father figure / mother figure	
indentifier #		

TOHO YA TABA:

TOHO YA TABA YA LIKEPE:

Libaka la toho ya taba ye?

LICINCEHO LISIKA EZAHAHALA KALE

Libaka la cinceho ye? Kisi kamani sene sisusuelize cinceho ye?

mushimani

Libaka Lelihanisa
1.
2.
3.

Libaka Lelilumeleza
1.
2.
3.

Libaka leo neni palezwi kuikambota nibona kuli lulumelelane

bo ndatahe

Libaka Lelihanisa
1.
2.
3.

Libaka Lelilumeleza
1.
2.
3.

Libaka leo neni palezwi kuikambota nibona kuli lulumelelane

bo mahe

Libaka Lelihanisa
1.
2.
3.

Libaka Lelilumeleza
1.
2.
3.

Libaka leo neni palezwi kuikambota nibona kuli lulumelelane

TOHO YA TABA YA LIKEPE:

Libaka la toho ya taba ye?

CINCEHO

Cinceho ye neiyazahalile cwani? Kisi kamani sene sitisize cwalo?

Kimani / Kina'i
Mukwa
Manzwi Amusu̇suezi
mushimani
Kalabo

musebezi waka

Kimani / Kina'i
Mukwa
Manzwi Amusu̇suezi
bo ndatahe
Kalabo

musebezi waka

Kimani / Kina'i
Mukwa
Manzwi Amusu̇suezi
bo mahe
Kalabo

musebezi waka

TOHO YA TABA YA LIKEPE:

Libaka la toho ya taba ye?

KACENU

Kin'i sene sitisize kuli lubasi lucince? Libaka?

mushimani

Muzamaisi / Mulumelezi

bo ndatahe

Muzamaisi / Mulumelezi

bo mahe

Muzamaisi / Mulumelezi

CHOLINGA CHA NKHANI:

A-tarian	Actively Aligning	Not Advocating
A-tative	Anticipating	Advocating
P-missive	young man / father figure / mother figure	
indentifier #		

CHAPAMUTU:

chifukwa chanicholinga ichi?

MUKALIBE MUCHINJA

Chifukwa chani munachinja? Kodi nichani china lengesa kapena kulimbisa?

Chifukwa Chani Ukana
1.
2.
3.

m'nyamata wachichepele

Chifukwa Chani M'vomela
1.
2.
3.

Chinalengesa kuti basavomekeze na ine

Chifukwa Chani Ukana
1.
2.
3.

atate / pena obaimilila

Chifukwa Chani M'vomela
1.
2.
3.

Chinalengesa kuti basavomekeze na ine

Chifukwa Chani Ukana
1.
2.
3.

amayi / pena obaimilila

Chifukwa Chani M'vomela
1.
2.
3.

Chinalengesa kuti basavomekeze na ine

CHAPAMUTU:

chifukwa chanicholinga ichi?

KUCHINJA

Munachinja bwanji? Chiyamba bwanji?

Ndania / Chani
Njila
Utenga Ochinja Maganizo

m'nyamata wachichepele

Zamene Na Chita

Mbalia yanga pazochitika

Ndania / Chani
Njila
Utenga Ochinja Maganizo

atate / pena obaimilila

Zamene Na Chita

Mbalia yanga pazochitika

Ndania / Chani
Njila
Utenga Ochinja Maganizo

amayi / pena obaimilila

Zamene Na Chita

Mbalia yanga pazochitika

CHAPAMUTU:

chifukwa chanicholinga ichi?

LERO

Chinalengesa kuti banja ichinje?
Chifukwa?

m'nyamata wachichepele

Osogolera / Ovomekeza

atate / pena obaimilila

Osogolera / Ovomekeza

amayi / pena obaimilila

Osogolera / Ovomekeza
