# Supplementary material for: Exploring joint decision-making and family dynamics to identify barriers and enablers for early adolescent medical circumcision (EAMC) uptake in Zambia for HIV prevention: An innovative methodology
Source: PLoS One. 2025 Apr 29;20(4):e0319472. doi: 10.1371/journal.pone.0319472 (PMC12040159; doi:10.1371/journal.pone.0319472)
Supplement: S3 File — (DOCX) [file pone.0319472.s003.docx]

**INTRODUCE AS A BROADER HEALTH INTERVIEW**

**Recruiter approaches Father/Mother/Guardian in the community and introduces the study.**

**Introduction and consent**

Hello. My name is _______________________________________. I am working with BRANDCOMM in collaboration with Ministry of Health & Bill and Melinda Gates Foundation. We are conducting a study to understand how health and hygiene decisions for sons are made in various households.

Your feedback will help The Ministry of Health design more appropriate health programs for your community.

The themes include the interactions between boys and parents, respective roles in decision making, interactions with the health system, circumcision choices etc.

| *We’d like to run through certain questions to recruit you for the study. Would you be interested to speak with us today?*  Yes - Continue  No - Thank and close |
| --- |

I have some questions am going to ask you. Your answers will be confidential. This interview will take about 15 minutes. All the answers you give will be confidential and will not be shared with anyone other than members of our survey team.

After answering the survey questions, I will let you know if you have qualified to participate in the next phase of the survey.

Do you have any questions? *(Interviewer give respondent an opportunity to ask questions)*

| 1. **CONSENT AGREEMENT** | | | |
| --- | --- | --- | --- |
| **Consent** | RESPONDENT AGREES RESPONDENT | 1 | **CONTINUE TO SECTION A** |
|  | DOES NOT AGREE TO BE INTERVIEWED | 2 | **THANK THE RESPONDENT AND TERMINATE** |

| 1. **IDENTIFICATION** | | | | | | | | |
| --- | --- | --- | --- | --- | --- | --- | --- | --- |
| **Place Name ____________________________**  **Name of the Household Head ____________________**   \| **Household Number …………………………….** \| \|  \|  \|  \|  \| \| --- \| --- \| --- \| --- \| \|  \|  \|  \|  \| \| \| --- \| --- \| --- \| --- \| --- \| --- \| --- \| --- \| --- \| --- \| | | | | | | | | |
|  | | | | | | | | |
| **Interviewer**  **Number** |  |  | **Team**  **Supervisor** | **Name _______________** | | | |  |
|  |  | |  | **Number** |  |  |  |  |

| A1 | How many males aged 10 -17 live in this household? | Age |
| --- | --- | --- |
|  | Name of HH member |  |
| 1 |  |  |
| 2 |  |  |
| 3 |  |  |
| 4 |  |  |
| 5 |  |  |
| 6 |  |  |

| 7. | What is the exact age of young males in this household aged 10-17? RECORD FOR ALL MALES FALLING WITHIN SURVEY AGE GROUPS | Boy 1- Record age __  Boy 2- Record age __  Boy 3- Record age __  Boy 4- Record age __ |
| --- | --- | --- |
| 8 | Parenting Style Identified (fill in the blank) |  |

**PROCEED IF THERE ARE MALES AGED 10-17; OTHERWISE THANK RESPONDENTS AND CLOSE THE INTERVIEW – OBSERVE AGE QUOTA**

| **PARENTING STYLE- HOUSEHOLD TYPE – ONLY ASK FATHER FIGURE** | | | | **SINGLE MENTION** |
| --- | --- | --- | --- | --- |
| P1  ASK ALL | How often…  Do you threaten to punish your son and then do not actually punish him? | Never | 1 |  |
|  |  | Almost Never | 2 |  |
|  |  | Sometimes | 3 |  |
|  |  | Often | 4 |  |
|  |  | Always | 5 |  |
|  | | | |  |
| P2  ASK ALL | How often …  Does your son inform you where he is going? | Never | 1 |  |
|  |  | Almost Never | 2 |  |
|  |  | Sometimes | 3 |  |
|  |  | Often | 4 |  |
|  |  | Always | 5 |  |
|  | | | |  |
| P3  ASK ALL | How often…  Does your son talk you out of being punished after he has done something wrong? | Never | 1 |  |
|  |  | Almost Never | 2 |  |
|  |  | Sometimes | 3 |  |
|  |  | Often | 4 |  |
|  |  | Always | 5 |  |
|  | | | |  |
| P4  ASK ALL | How often…  Does your son stay out in the evening after the time he is supposed to be home? | Never | 1 |  |
|  |  | Almost Never | 2 |  |
|  |  | Sometimes | 3 |  |
|  |  | Often | 4 |  |
|  |  | Always | 5 |  |
|  | | | |  |
| P5  ASK ALL | How often…  Is your son out with friends you don’t know? | Never | 1 |  |
|  |  | Almost Never | 2 |  |
|  |  | Sometimes | 3 |  |
|  |  | Often | 4 |  |
|  |  | Always | 5 |  |
|  | | | |  |
| P6  ASK ALL | How often…  Do you let your son out of a punishment early (e.g. leaves punishment restrictions earlier than you originally said)? | Never | 1 |  |
|  |  | Almost Never | 2 |  |
|  |  | Sometimes | 3 |  |
|  |  | Often | 4 |  |
|  |  | Always | 5 |  |
| **CHECK QUOTAS AS PER CLASSIFICATION BELOW & RECRUIT; OTHERWISE PROCEED** | | | | |

***TOTAL THE POINTS IN PARENTHESIS TO DETERMINE CLASSIFICATION OR CONTINUE:***

Greater 23 – PERMISSIVE

*22 – 13 CONTINUE*

12–11 *AUTHORITARIAN*

| **PARENTING STYLE- HOUSEHOLD TYPE – ONLY ASK FATHER FIGURE** | | | |  |
| --- | --- | --- | --- | --- |
| P6  ASK ALL | How often…  Do you let your son know when he is doing a good job with something? | Never | 1 |  |
|  |  | Almost Never | 2 |  |
|  |  | Sometimes | 3 |  |
|  |  | Often | 4 |  |
|  |  | Always | 5 |  |
|  | | | |  |
| P8  ASK ALL | How often…  Do you compliment your son after he has done something well? | Never | 1 |  |
|  |  | Almost Never | 2 |  |
|  |  | Sometimes | 3 |  |
|  |  | Often | 4 |  |
|  |  | Always | 5 |  |
|  | | | |  |
| P9  ASK ALL | How often…  Do you praise your son if he behaves well? | Never | 1 |  |
|  |  | Almost Never | 2 |  |
|  |  | Sometimes | 3 |  |
|  |  | Often | 4 |  |
|  |  | Always | 5 |  |
| **CHECK QUOTAS AS PER CLASSIFICATION BELOW & RECRUIT** | | | | |

***TOTAL THE POINTS IN PARENTHESIS TO DETERMINE CLASSIFICATION OR TERMINATE:***

Above 10 – AUTHORITATIVE 1-9 TERMINATE

**SCREENEER FOR ADVOCATING & NOT ADVOCATING**

| 1. **STAGING SCREENER - AFFINITY TOWARDS VMMC – ASKED TO FATHER FIGURE ONLY** | | | | | |
| --- | --- | --- | --- | --- | --- |
| B1  ASK ALL | Thinking about “medical circumcision”, are you familiar with circumcision that is usually conducted in health care facilities/hospital? | | Yes | 1 | Continue |
|  |  |  | No | 2 | Terminate |
|  | | | | |  |
| B2 ASK ALL | Is your son aged ---- years been medically circumcised? | | *Yes* | 1 | Continue |
|  |  |  | *No* | 2 | Skip to B5 |
|  | | | | |  |
| B3 ASK ALL | When was your son circumcised? | | *Less than 6 months ago* | 1 | Proceed for Advocating  Proceed for Not Advocating |
|  |  | | *6 – 12 months ago* | 2 |  |
|  |  | | *More than 12 months ago* | 3 | Terminate |
| B4 ASK ALL | Which of the following statements are true for you or your family after your son got circumcised | *We were previously not willing to discuss our MC experience with other people/families but now are discussing* | | 1 | Recruit as Advocating  Recruit as Not Advocating |
|  |  | *At least one of our family members is not willing to discuss MC with other people/families* | | 2 |  |
| **Skip to QB10** | | | | | |

**SCREENEER FOR ACTIVELY ALIGNING & ANTICIPATING**

| 1. **STAGING SCREENER - AFFINITY TOWARDS VMMC – ASKED TO FATHER FIGURE ONLY** | | | | | |
| --- | --- | --- | --- | --- | --- |
| B1  ASK ALL | Thinking about “medical circumcision”, are you familiar with circumcision that is usually conducted in health care facilities/hospital? | Yes | 1 | | Continue |
|  |  | No | 2 | | Terminate |
|  | | | | |  |
| B2 ASK ALL | Is your son aged ---- years been medically circumcised? | *Yes* | 1 | | Terminate |
|  |  | *No* | 2 | | Proceed |
| B3 ASK THOSE WHO SAID NO AT B2 | Does anyone in your family want him to be circumcised? | *Yes* | | 1 | Proceed to B6  Terminate |
|  |  | *No* | | 2 |  |

|  |  |  | For | Against |
| --- | --- | --- | --- | --- |
| B4 | If yes, who in your family is for/against circumcision? | *Mother* | 1 | 1 |
|  |  | *Father* | 2 | 2 |
|  |  | *Son* | 3 | 3 |
|  |  | *Other (SPECIFY)* |  |  |
|  | Proceed if at least one family member is for VMMC | | | |

| B5 | Which of the following statements best describe your current position on VMMC (READ OUT) | *Our family has made up mind on when to circumcise and are talking about the right time to do it* | 1 | Recruit as Anticipating  Check quota and proceed |
| --- | --- | --- | --- | --- |
| B6 |  | *Our family has not made up mind on when to circumcise and are still talking about it* | 2 | Recruit as Actively Aligning  Check quota and proceed |
| **CHECK QUOTA ALLOCATION** | | | | |

| **ASK ALL** | | | |
| --- | --- | --- | --- |
| C1 | Who in your household makes final decision for the family? | *Mother* | 1 |
|  |  | *Father* | 2 |
|  |  | *Other (specify)* | 3 |

| **ASK ALL SCHOOLING STATUS** | | | |  |
| --- | --- | --- | --- | --- |
| D1 | Is your son currently attending school on a regular basis? | Yes | 1 | Recruit as **In school** |
|  |  | No | 2 | Proceed to D2 |

| D2 | What is the current status of your son’s school attendance? | *Used to attend, but has no intention of going back to school* | 1 | Recruit as **Out of school**  Check quota and proceed |
| --- | --- | --- | --- | --- |
|  |  | *Used to attend, stopped briefly but intend to continue in the next 3 months* | 2 | Terminate |
|  |  | *Has never attended school* | 3 | Recruit as **Out of school**  Check quota and proceed |
| **CHECK QUOTAS FOR OUT OF SCHOOL AND IN SCHOOL** | | | | |

| **ASK ALL Head of Household Demographics** | | | |
| --- | --- | --- | --- |
| D3  ASK ALL | Which of the following currently applies to the head of the household? | Married/living together | 1 |
|  |  | *Divorced/separated* | 2 |
|  |  | *Widowed* | 3 |
|  |  | *Single/never married* | 4 |
|  | | | |
| D4  ASK ALL | Sex  INT: OBSERVE | *Male* | 1 |
|  |  | *Female* | 2 |
|  | | | |
|  | | | |
| D5  ASK ALL | What is the highest level of education the head of the household has achieved? | *No formal education* | 1 |
|  |  | *Primary* | 2 |
|  |  | *Secondary* | 3 |
|  |  | Tertiary | 4 |
|  |  | *Other (specify) …….* | 6 |
|  |  | *Don’t know/refuse* | 8 |
|  | | | |

| D6  ASK ALL | Type of Residence | *Urban* | 1 |
| --- | --- | --- | --- |
|  |  | *Rural* | 2 |
|  | | | |

| D7  ASK ALL | Language | *Nyanja* | 1 |
| --- | --- | --- | --- |
|  |  | *Lozi* | 2 |
|  |  | *Other (Specify)* | 3 |
|  | | | |
| D8  ASK ALL | Religion | *Christian* | 1 |
|  |  | *Muslim* | 2 |
|  |  | *Customary* | 3 |
|  |  | *Other (Specify)* | 4 |
|  | | | |
| D9  ASK ALL | Employment status | *Not employed* | 1 |
|  |  | *Employed for cash* | 2 |
|  |  | *Employed not for cash* | 3 |
|  |  | *Don’t know/refuse* | 8 |
|  | | | |
| D10  ASK ALL | Occupation (please specify) | *…………………………………………………….* |  |
|  |  | *Don’t know/refuse* | 8 |

| **SIGNING OF CONSENT FORMS AND INVITATION TO PARTICIPATE IN THE SURVEY** |
| --- |
