# Supplementary material for: Exploring joint decision-making and family dynamics to identify barriers and enablers for early adolescent medical circumcision (EAMC) uptake in Zambia for HIV prevention: An innovative methodology
Source: PLoS One. 2025 Apr 29;20(4):e0319472. doi: 10.1371/journal.pone.0319472 (PMC12040159; doi:10.1371/journal.pone.0319472)
Supplement: Attachment — Submitted filename: Dynamics of VMMC decisionin Zambia - Reviewers comments.docx [file pone.0319472.s007.docx]

**TITLE:** A qualitative research methodology to understand the drivers of the decision dynamic among adolescent boys and their parents/guardians affecting uptake of voluntary medical male circumcision for HIV prevention in Zambia

**REVIEWER’S COMMENTS**

The manuscript contains very crucial and novel information in the VMMC space. To further enhance the manuscript, the following are some itemized comments followed by the overall comment and recommendation

A. **ITEMISED COMMENTS**

**Title:**

The title needs refinement and to be made concise. For example, mentioning the methodology in the title may not be necessary.

**Introduction and background:**

Authors managed to articulate very well the complexity and dynamics of decision making regarding VMMC among adolescents. However:

- The knowledge gap (statement of the problem) which this study intends to address or close is not clearly defined.
- The purpose and objectives of the study are also note clearly stated.

**Methodology:**

The method sounds robust, procedural and well thought. The section may, however, benefit from some further refinements either for clarity’s sake or adding to the credibility of the content. Examples include the following:

- Lines 177 – 178 “… the insights gained from a previous study of adults aged 18 and over …”. Consider stating the reference for the previous study.
- Similarly, the concept of journey mapping was well described (Line 233-239). However, consider backing up the facts with relevant literature.
- Consider adding details which answer the following questions:
- Out of the entire district(s), where exactly was the sampling done, and why or how?
- Which schools and/or health facilities were targeted, and why or how?
- How were the sample sizes determined?
- Was there any link between the sampled adolescents and the parents/guardians?
- Was there some deliberate effort to stratify and ensure representation of certain categories, or it was all by coincidence
- Line 225 talks of transcripts being analysed per **household**. What was the sampling unit for this phase of the study, was it a household or individuals? Were the participants also interviewed as a household or individually or both?
- Line 220-223 talk about interview tool, structured around objectives and themes, but apparently these themes and objectives were not stated anywhere.

*** These are observations and probes just for the immersion aspect of the method. Most of them, plus more, apply as well for the journey mapping and ethnolab phases

**Results and discussion**

Appreciable and commendable effort was made to present results with illustrative quotations. However:

- The large volumes of data and the absence of a clear purpose and objectives of the study makes the structure of this section difficult to follow.
- The discussion is not well grounded in literature.

**Conclusion**

- In the absence of clear purpose and objectives from the start, this section becomes hanging.
- Instead of describing the contributions of the different elements of the methodology, it would be more appropriate to give an overview of the key findings, and direct answers to the initial research questions.

**Other concerns**

- Participants were given incentives (lines 207 - 209), and that is often regarded as coercive and hence unethical.
- Language editing is also recommended for this submission

B. **OVERALL IMPRESSION AND RECOMMENDATION.**

- There seems to be too much content compressed into this manuscript, making it difficult to exhaust refined descriptions of each methodological aspect. Thus, there are some gaps in the methodological descriptions.
- I would recommend that authors consider splitting the manuscript into 2 or 3 sizeable and refined manuscripts. For example, each of the 3 elements: the interviews, journey mapping and ethnolab can constitute a distinct manuscript, where all the refined details are included, along with rationales
