# Supplementary material for: Exploring joint decision-making and family dynamics to identify barriers and enablers for early adolescent medical circumcision (EAMC) uptake in Zambia for HIV prevention: An innovative methodology
Source: PLoS One. 2025 Apr 29;20(4):e0319472. doi: 10.1371/journal.pone.0319472 (PMC12040159; doi:10.1371/journal.pone.0319472)
Supplement: Attachment — Submitted filename: Final - Rebuttal Letter.docx [file pone.0319472.s008.docx]

1. Is the manuscript technically sound, and do the data support the conclusions? 
 
The manuscript must describe a technically sound piece of scientific research with data that supports the conclusions. Experiments must have been conducted rigorously, with appropriate controls, replication, and sample sizes. The conclusions must be drawn appropriately based on the data presented.

Reviewer #1: Partly

Reviewer #2: Partly

Reviewer #3: Partly

2. Has the statistical analysis been performed appropriately and rigorously?

Reviewer #1: N/A

Reviewer #2: N/A

Reviewer #3: N/A

3. Have the authors made all data underlying the findings in their manuscript fully available? 
 
The [PLOS Data policy](http://www.plosone.org/static/policies.action#sharing) requires authors to make all data underlying the findings described in their manuscript fully available without restriction, with rare exception (please refer to the Data Availability Statement in the manuscript PDF file). The data should be provided as part of the manuscript or its supporting information, or deposited to a public repository. For example, in addition to summary statistics, the data points behind means, medians and variance measures should be available. If there are restrictions on publicly sharing data—e.g. participant privacy or use of data from a third party—those must be specified.

Reviewer #1: No

Reviewer #2: Yes

Reviewer #3: No

4. Is the manuscript presented in an intelligible fashion and written in standard English? 
 
PLOS ONE does not copyedit accepted manuscripts, so the language in submitted articles must be clear, correct, and unambiguous. Any typographical or grammatical errors should be corrected at revision, so please note any specific errors here.

Reviewer #1: Yes

Reviewer #2: No

Reviewer #3: No

5. Review Comments to the Author

Please use the space provided to explain your answers to the questions above. You may also include additional comments for the author, including concerns about dual publication, research ethics, or publication ethics. (Please upload your review as an attachment if it exceeds 20,000 characters)

**Reviewer #1:**

Lessons from the investigations presented in the paper are helpful and this deep dive into the drivers and barriers to circumcision is informative. Insightful lessons are presented in the results section but there is lack of linkage with the methods and the data; that is, it was not clear how or from where the categories were derived (interviews, journey mapping, and/or ethnolab?). Nonetheless, with some revision, this research should be published somewhere.

Here are comments about specific sections/content:

- The authors claimed that VMMC presents the greatest possible protection but does not specify against what. It is safe to assume against HIV. What about PrEP and condoms?
  - **Rebuttal:** Edited to address feedback and introduce clarity in communication
  - **Lines: 47 - 54**
  - **Edits:** VMMC is a one-time intervention, unlike Pre-exposure prophylaxis (PrEP) or condoms, that significantly reduces the risk of HIV transmission and other sexually transmitted infections (STIs ) [1,2,3]. It is particularly relevant for adolescents as the procedure offers the greatest protection if it is done before sexual debut, as it reduces the lifetime risk of acquiring HIV. Therefore, targeting adolescent boys who have not yet become sexually active maximizes the health benefits of VMMC. VMMC is relevant for adolescents because it is a one-time intervention that confers the greatest possible protection if it takes place before their sexual debut.
- Specify for whom the 15 VMMC countries are priority (WHO, UNAIDS, PEPFAR?)
- **Rebuttal:** Edited to include requested information
- **Lines: 44**
- **Edits:** VMMC has been a key part of HIV prevention interventions in 15 priority countries in Southern and Eastern Africa as identified by UNAIDS & WHO [4].
- The statement, “VMMC is relevant for adolescents because it is a one-time intervention that confers the greatest possible protection if it takes place before their sexual debut” is misleading. VMMC does not provide the “greatest possible protection” but it is the only one-time intervention with partial protection since other prevention methods like condoms and PrEP require consistent use to be effective.
  - **Rebuttal:** Edited to address feedback and introduce clarity in communication
  - **Lines: 47 - 54**
  - **Edits:** VMMC is a one-time intervention, unlike Pre-exposure prophylaxis (PrEP) or condoms, that significantly reduces the risk of HIV transmission and other sexually transmitted infections (STIs ) [1,2,3]. It is particularly relevant for adolescents as the procedure offers the greatest protection if it is done before sexual debut, as it reduces the lifetime risk of acquiring HIV. Therefore, targeting adolescent boys who have not yet become sexually active maximizes the health benefits of VMMC. VMMC is relevant for adolescents because it is a one-time intervention that confers the greatest possible protection if it takes place before their sexual debut.
- A few misplaced commas for lines 50-54 make the sentence difficult to follow.
  - **Rebuttal:** Edited to break up sentence into shorter, more concise sentences to improve clarity in communication
  - **Lines: 58 - 63**
  - **Edits:** In 2020 the World Health Organization (WHO) revised the age criteria for VMMC programs for adolescents (also referred to as Early Adolescent Male Circumcision) to 15-19 years [6] because of concerns about 10-14 year old adolescents’ ability to give informed assent to a medical procedure that is non-urgent and irreversible and due to a higher rate of serious adverse events following Male Circumcision (MC) among this age group. In line with this updated guideline, Zambia’s VMMC program now focuses on reaching uncircumcised males aged 15 and above [7].
- Additionally, the statement that, “Since 2020 the World Health Organization (WHO) has recommended that VMMC programs for adolescents focus on those aged 15-19, because of concerns about a higher rate of some serious adverse events among adolescents ages 10-14 years,” needs citation.
  - **Rebuttal:** Citations added for edited text
  - **Lines: 59 and 63**
  - **Edits:** Same citation for whole paragraph - [Reference #6 in paper - World Health Organization. Policy brief: preventing HIV through safe voluntary medical male circumcision for adolescent boys and men in generalized epidemics: recommendations and key considerations. Geneva: The Organization; 2020.]
- 72 The authors use a “behavioral decision making framework” but a brief description is needed about this framework because it seems to be the foundation of this paper.
  - **Rebuttal:** We have edited the manuscript to provide clarity regarding the objectives of this paper, which is to describe the qualitative methodology undertaken by the authors that aimed to address challenges that traditional qualitative research faces, especially in the context of EAMC decision making. While the behavioural decision making framework was the output of our research, detailing it out falls out of the scope of this paper (as outlined in **Line 503**).
  - **Lines: 73 - 84**
  - **Edits:** The present paper describes a methodology specially designed to address the complexity underlying decision-making related to voluntary medical male circumcision (VMMC) for adolescents with their parents/guardians in Zambia. By focusing on the joint decision-making dynamics within families and identifying the drivers and barriers to circumcision for adolescents aged 10-19, our methodology provided unique insights into how interventions could be more effectively tailored to increase VMMC uptake. Our study included younger adolescents (10-14 years in addition to 15-19 years) as the study design and initial fieldwork was completed prior to Zambia’s alignment with the revised WHO guidelines promoting VMMC for those aged 15 and older [6]. This paper details the theoretical foundations and qualitative research methods we employed, highlighting how these approaches successfully addressed limitations in traditional research methodologies, ultimately providing actionable insights and a behavioral decision-making framework for improving VMMC uptake (a more detailed account of which will be published separately).
- 132 Add citation to, “Decisions are driven by emotions, by mental shortcuts (heuristics) and other non-conscious drivers.”
  - **Rebuttal:** Citation added
  - **Lines: 150**
  - **Edits:** [27] - Lerner JS, Li Y, Valdesolo P, Kassam KS. Emotion and Decision Making. Annual Review of Psychology [Internet]. 2015;66(1):33.1–25. Available from: <https://www.annualreviews.org/content/journals/10.1146/annurev-psych-010213-115043>]
- A better description of Immersion method is needed. It sounds like interviews but this isn’t directly stated. If this is the case, why not just label it interviews? The analytical method for interview data was unclear. It sounds like the stages derived were based on previous experience and from findings from the current interviews but how the Immersion findings supported stages derived from “previous experience” was unclear.
- **Rebuttal:**

1. We have re-named "immersion" as "formative research" across the document, and have called out that it consisted of semi-structured in-depth interviews

2. We have included additional information on our analysis method for the formative research

3. We have included text to inform the linkage between findings from previous research and the formative research

- **Edits:**

1. **Lines 194 - 197** – 1. The formative research consisted of semi-structure in-depth interviews with adolescent boys aged 10-17 (hereafter referred to as younger adolescents) that, along with previous research undertaken by the consortium [Vaish et al., 2015], informed the design of the journey mapping and Ethnolab.

2. **Lines 269 – 277** - 2. Transcripts of the interviews were analyzed using deductive analysis, drawing from Ellsworth & Scherer's emotional appraisal theory [43] and thematic learnings from our previous research [39]. The authors (Namiya Jain, Rasi Surana and Alok Gangaramany) coded transcripts to systematically identify key themes and appraisal dimensions emerging from the data. Data from each household were organized and analyzed together, allowing us to examine interviews across all members of a single household in context of one another. This approach enabled more effective triangulation of insights. After coding, the data were synthesized by respondent category and further grouped by age band to capture nuanced patterns across different demographic groups.

3. **Lines 278 - 281** - 3. The formative research adapted hypotheses from the previous VMMC research for the joint decision-making context of EAMC to arrive at the stages of a households journey to EAMC (Table 4). This was used as a basis for further stages of research in the study.

- At the beginning of the Methods section, brief explanation of Immersion, Journey Mapping, Ethnolab is needed. It was difficult to follow the authors’ point about each of these because the description did not come until later in the paper.
- **Rebuttal:** We have included a table detailing out the time frame, sample and objectives of each phase of research to help improve understanding and readability of this section
- **Lines: 224**
- **Edits:** Table 2
- Time orientation for each of the data collection methods is needed. When were data for Immersion, Journey Mapping, Ethnolab collected and how long did it take ?
- **Rebuttal:** The time orientation for each phase of research has been introduced in the process overview table
- **Lines: 224**
- **Edits:** Table 2
- The authors “hypothesized that household decision-making dynamics would vary based on the dominant parenting style within the household” but not much background justification was presented on this “hypothesis.” Additionally, the authors used the Alabama Parenting Questionnaire to assess parenting style and stated to have “contextualized to the Zambian community context.” A description of how they “contextualized” this measure is needed (e.g., back translated, revised the items?).
- **Rebuttal:** Details added to address feedback
- **Edits:**

1. **Lines: 323 – 326 -** As decision-making around adolescent health combines factors like interactions between parents and adolescents, family values/rules, and roles, the prior science around parenting styles was a useful lens to understand the existing context of the household as a unit [47].

2. **Lines: 345 - 349 -** We took three steps in adapting the parenting style survey to the Zambian context: 1. We highlighted phrases we judged as unique in the American (school) context and developed alternative phrasing more suitable for the African/Zambian context. 2. Our local research lead in Zambia re-articulated and translated questions into local context and language. 3. Questions were stress tested in a limited sample pilot study.

- The analytical plan needed more details (e.g., number of coders and their background, software used for analysis, the role of the authors). It would be helpful for authors to go through the Consolidated criteria for reporting qualitative studies (COREQ) to ensure that critical information have been presented.
- **Rebuttal:** Details added across all research stages to address feedback
- **Edits:**

1. **Lines 269 – 277 -** Transcripts of the interviews were analyzed using deductive analysis, drawing from Ellsworth & Scherer's emotional appraisal theory [add citation] and thematic learnings from our previous research [add citation]. The authors (Namiya Jain, Rasi Surana and Alok Gangaramany) coded transcripts to systematically identify key themes and appraisal dimensions emerging from the data. Data from each household were organized and analyzed together, allowing us to examine interviews across all members of a single household in context of one another. This approach enabled more effective triangulation of insights. After coding, the data were synthesized by respondent category and further grouped by age band to capture nuanced patterns across different demographic groups.

2. **Lines 362 – 369 -** Tim Sweeney and Jeff Mulhausen (authors) were primary coders for the data. Each has been deploying the underlying methodology for over 10 years. Initially, notes and transcripts were organized and translated by local moderators to interpret and preserve context. The coders then took a thematic analysis approach to analyze the data. Each coder independently coded interviews. After the initial coding round, coders collectively reviewed preliminary codes to refine the framework, address discrepancies and capture key themes. Once the set of codes were finalized, the coders re-coded the interviews to ensure reliability and consistency.

3. **Lines 457 – 463 -** The transcripts were analyzed using deductive analysis, drawing from Ellsworth & Scherer's emotional appraisal theory [43] and theme matching and pattern identification from our previous research [Vaish et al., 2015]. The authors (Namiya Jain, Rasi Surana and Alok Gangaramany) completed an initial coding of transcripts to systematically identify key themes and dimensions emerging from the data. The dimensions mapped were barrier themes, context, and emotional appraisal elements. The codes from each stage were further analyzed as separate groups.

- This paper mentioned multiple times the triad (i.e., adolescent, mother, father). Was there ever a case in which the unit was not a triad or had different members (single parent households or non-parent caregiver). If not, how might this lack of representation limit the generalizability of the findings to certain types of families?
- **Rebuttal:** Clarification about the inclusion of non-parent caregivers and single parent households included. Furthermore, to streamline language across the document, references of son and parents/guardians have been changed to adolescent and caregivers.
- **Lines: 211 - 213**
- **Edits:** Across the 3 phases of research, data was collected from adolescents and caregivers (including parent and non-parent caregivers and single-parent households) to understand the joint-decision making dynamics at play for EAMC decisions.

**Reviewer #2:**

This paper requires some significant work to pass for publication. Here are my key comments:

- Methods:
- 1) The methods mention immersions as a method and describe this as in-depth interviews. This is incorrect. Immersions are a lot of in-depth and include spending time in participant lives. This should be corrected.
- **Rebuttal:** We have re-named "immersion" as "formative research" across the document, and have called out that it consisted of semi-structured in-depth interviews
- **Lines: 194 – 197**
- **Edits:** The formative research consisted of semi-structure in-depth interviews with adolescent boys aged 10-17 (hereafter referred to as younger adolescents) that, along with previous research undertaken by the consortium [39], informed the design of the journey mapping and Ethnolab.
- 2) Further the recruitment criteria should separate adolescent boys from parents since parents do not really fit into circumcision status.
- **Rebuttal:** We have re-worded language around this to clarify that recruitment was completed based on adolescents circumcision status alone, and not the parents. The confusion arose from some errors in sentence construction in the original document.
- **Lines: 227 - 229**
- **Edits:** A sample of 16 adolescent boys aged 10-17 and 22 caregivers were recruited from Lusaka and Chongwe districts for interviews. They were chosen based on the adolescent’s circumcision status, age, and school enrollment status (Table 3).
- 3) Also, you further segment parents in parenting style but you do not mention this as a criteria for recruitment of parents.
- **Rebuttal:** Participants were screened based on parenting styles only in the journey mapping phase of research (**Line 332, Table 5**). This is because journey mapping sought to identify barriers and enablers of EAMC decision-making, which we had hypothesized is influenced by the dominant parenting style within a household (**Lines 320 – 323**). On the other hand, the Ethnolab phase was designed to identify the levers to overcome barriers to EAMC decision-making. While the levers being tested were based on the enablers and barriers identified in the journey mapping phase, we did not wish to limit the utility of the levers by making them specific to the parenting styles. This means that we did not recruit by parenting style for the Ethnolab, but did collect this data to ensure representation. While the levers were kept parenting-style agnostic to make it more actionable, we did analyse data to see if there were key differences in how caregivers with different parenting styles were responding to the levers tested. However, we found this not to be the case.
- **Edits:** No edits made to the manuscript
- 4) Lastly, given that decision making reduces with age, should the study not have interviewed more of the younger ages i.e. below 15 years?
- **Rebuttal:** Given that caregiver involvement is legally necessitated for all adolescents aged 17 years and below, there is increased parental involvement in EAMC decision-making for these adolescents. As a result, we categorized adolescents into 2 age bands: younger adolescents aged between 10-17 years, and older adolescents aged between 18-19 years. While the immersion and journey mapping phases exclusively focused on the younger adolescent cohort, older adolescents were only included in the Ethnolab phase of research. However, as we noted in **Lines 769 - 773**, there is fairly high parental involvement even among older adolescents despite not requiring legal consent. For this reason, we believe that our sample distribution in this case is justified.
- **Edits:** No edits made to the manuscript
- For ethics approval it standard to mention approval number if this is provided by the board used.
- **Rebuttal:** We have now included this in the manuscript
- **Lines: 214 - 216**
- **Edits:** Ethical approval was received from the ERES Converge review board (Approval number: 2020-Jan-019) in Zambia before each stage of the research, and from Zambia’s National Health Research Authority, for both adolescents aged 10-17 and adolescents aged 18-19.
- The work proposes to proffer a behavioral decision-making framework. This is not given in the results. This is a key outcome of this work and due to this weakness this work is falling short or may need to be recasted.
- **Rebuttal:** We have edited the manuscript to provide clarity regarding the objectives of this paper, which is to describe the qualitative methodology undertaken by the authors that aimed to address challenges that traditional qualitative research faces, especially in the context of EAMC decision making. While the behavioural decision-making framework was the output of our research, detailing it out falls out of the scope of this paper (as outlined in **Line 503**).
- **Lines: 73 - 84**
- **Edits:** The present paper describes a methodology specially designed to address the complexity underlying decision-making related to voluntary medical male circumcision (VMMC) for adolescents with their caregivers in Zambia. By focusing on the joint decision-making dynamics within families and identifying the drivers and barriers to circumcision for adolescents aged 10-19, our methodology provided unique insights into how interventions could be more effectively tailored to increase VMMC uptake. Our study included younger adolescents (10-14 years in addition to 15-19 years) as the study design and initial fieldwork was completed prior to Zambia’s alignment with the revised WHO guidelines promoting VMMC for those aged 15 and older [6]. This paper details the theoretical foundations and qualitative research methods we employed, highlighting how these approaches successfully addressed limitations in traditional research methodologies, ultimately providing actionable insights and a behavioral decision-making framework for improving VMMC uptake (a more detailed account of which will be published separately).
- Related to above, the results section and the title do not fully align. Results focus on barriers and less on decision making. While barriers a underlying to decisions this linkage is not fully examined. The study then becomes more of a traditional barriers and motivators to circumcisions. These are well documented in literature and thus the study will not be adding new knowledge. This linkage needs to be established for the work to be publishable. I find this to be a key comment of my review.
- **Rebuttal:** In order to address these concerns, we have briefly introduced our behavioural decision framework. In doing so, we present the barriers and drivers in the context of an EAMC decision-making journey thereby showcasing how our methodology helped uncover new knowledge.
- **Lines: 489**
- **Edits:** Table 9

**Reviewer #3:**

The manuscript contains very crucial and novel information in the VMMC space. To further enhance the manuscript, the following are some itemized comments followed by the overall comment and recommendation

**A.**	**ITEMISED COMMENTS**

**Title:**

- The title needs refinement and to be made concise. For example, mentioning the methodology in the title may not be necessary.
  - **Rebuttal:** Noted, and edits made
  - **Lines: 1 - 6**
  - **Edits:**

1. Long Title - Exploring Joint Decision-Making and Family Dynamics to Identify Barriers and Enablers for Early Adolescent Medical Circumcision (EAMC) Uptake in Zambia for HIV Prevention: An Innovative Methodology

2. Short Title - Joint Decision-Making for Adolescent Circumcision in Zambia: An Innovative Methodology

**Introduction and background:**

Authors managed to articulate very well the complexity and dynamics of decision making regarding VMMC among adolescents. However:

- The knowledge gap (statement of the problem) which this study intends to address or close is not clearly defined.
  - **Rebuttal:** We have edited the introduction and background section to provide clarity regarding the statement of the problem in this paper
  - **Lines:** **Multiple between 64 and 189**
  - **Edits:** Multiple
- The purpose and objectives of the study are also not clearly stated.
  - **Rebuttal:** We have edited the manuscript to provide clarity regarding the objectives of this paper, which is to describe the qualitative methodology undertaken by the authors that aimed to address challenges that traditional qualitative research faces, especially in the context of EAMC decision making.
  - **Lines: 73 – 84**
  - **Edits:** The present paper describes a methodology specially designed to address the complexity underlying decision-making related to voluntary medical male circumcision (VMMC) for adolescents with their parents/guardians in Zambia. By focusing on the joint decision-making dynamics within families and identifying the drivers and barriers to circumcision for adolescents aged 10-19, our methodology provided unique insights into how interventions could be more effectively tailored to increase VMMC uptake. Our study included younger adolescents (10-14 years in addition to 15-19 years) as the study design and initial fieldwork was completed prior to Zambia’s alignment with the revised WHO guidelines promoting VMMC for those aged 15 and older [6]. This paper details the theoretical foundations and qualitative research methods we employed, highlighting how these approaches successfully addressed limitations in traditional research methodologies, ultimately providing actionable insights and a behavioral decision-making framework for improving VMMC uptake (a more detailed account of which will be published separately).

**Methodology:**

The method sounds robust, procedural and well thought. The section may, however, benefit from some further refinements either for clarity’s sake or adding to the credibility of the content. Examples include the following:

- Lines 177 – 178 “… the insights gained from a previous study of adults aged 18 and over …”. Consider stating the reference for the previous study.
  - **Rebuttal:** Citation added
  - **Lines: 206**
  - **Edits:** [39] - Vaish A, Sgaier S, Kretschmer S, Prasad R, Sweeney T, Mulhausen J, et al. Belief, Intent, Action,… Action! Bridging Intent – Action Gap: How Behavioural Economics and Consumer Journey Mapping Explains Decision Making to Identify Levers to Impel Consumer Action. In: ESOMAR Publication Series. Amsterdam: ESOMAR; 2015.
- Similarly, the concept of journey mapping was well described (Line 233-239). However, consider backing up the facts with relevant literature.
- **Rebuttal:** Relevant literature has been added
- **Lines:** **285 - 288**
- **Edits:** Journey mapping is a research methodology that analyzes the actual and/or ideal experience of people who have fully or partially achieved a behavioral outcome [44]. It is participatory in nature It is designed to counter the “say-do” gap through the act of making: the respondent makes their own journey artifact over the course of a journey mapping interview [39].
- Consider adding details which answer the following questions:
  - Out of the entire district(s), where exactly was the sampling done, and why or how?
- **Rebuttal:** Details added on which districts were sampled and why those districts were chosen
- **Lines: 197 - 199**
- **Edits:** Sampling for these phases was done primarily in 2 districts - the Western District of Sioma and Lusaka district, due to their rural, non-circumcising tradition (Sioma) and urban and cosmopolitan cultural characteristics (Lusaka) respectively.
  - Which schools and/or health facilities were targeted, and why or how?
- **Rebuttal:** Details added on how study sites were identified
- **Lines: 202**
- **Edits:** Table 1
  - How were the sample sizes determined?
    - **Rebuttal:** Explanation added on how sample sizes were determined
    - **Edits:**

1. **Lines – 229 - 235** – Formative Research - In studies utilizing qualitative methods, it is recommended that sample sizes be kept small to facilitate in-depth analysis of participant responses [40]. The sample size for this phase therefore, whilst chosen to be large enough to collect extensive and nuanced information on factors influencing EAMC, was limited to under 40 to allow detailed exploration. Additionally, the concept of 'informational redundancy' or 'data saturation', in which ‘gathering fresh data no longer sparks new theoretical insights’ [41], proved instrumental in assessing the adequacy of sample size.

2. **Lines 316 - 318** - Journey Mapping - This sample size was determined in accordance with previous studies in healthcare research that made use of journey mapping, which have been found to have recruited approximately 71 to 100 participants [45] as was done in the present study.

3. **Lines 382 - 384** - Ethnolab - This sample size was found to consistent with existing literature which have used mixed-methods [49] and gamification [50] to study HIV prevention, thereby enhancing the credibility of the findings and allowing for a balance between quantitative rigor and qualitative depth.

- Was there any link between the sampled adolescents and the parents/guardians?
- **Rebuttal:** This information is already included in the manuscript. In the formative research and journey mapping phase, households were recruited as a unit comprising of 3 respondents - an adolescent male and his parents/guardians. Hence, a link existed between them in these phases of the study. While the data collection happened at the level of each individual within the household, data was aggregated at the level of each household for analysis across the 2 phases (referenced in **Lines 272 - 274** (formative research) and **Line 358** (journey mapping)). No link existed between the sampled adolescents and parents/guardians in the ethnolab phase, however. This was because the Ethnolab was run as FGDs with adolescents and caregivers separately to understand levers for behaviour change. This didn't require us to organise and analyse data by households.
- **Edits:** No edits made to the manuscript
- Was there some deliberate effort to stratify and ensure representation of certain categories, or it was all by coincidence
- **Rebuttal:**

1. The present study used non-probability purposive sampling, hence stratification of the sample was not possible.

2. However, accuracy through representation was ensured by the inclusion of single parents and non-parent guardians in the sample for all research phases.

- **Lines: 211 - 213**
- **Edits:**

1. No edits

2. Across the 3 phases of research, data was collected from adolescents and caregivers (including parent and non-parent caregivers and single-parent households) to understand the joint-decision making dynamics at play for EAMC decisions.

- Line 225 talks of transcripts being analysed per **household**. What was the sampling unit for this phase of the study, was it a household or individuals? Were the participants also interviewed as a household or individually or both?
- **Rebuttal:** This information is already included in the manuscript. In the formative research and journey mapping phase, households were recruited as a unit comprising of 3 respondents - an adolescent male and his parents/guardians. Hence, a link existed between them in these phases of the study. While the data collection happened at the level of each individual within the household, data was aggregated at the level of each household for analysis across the 2 phases (referenced in **Lines 272 - 274** (formative research) and **Line 358** (journey mapping)). No link existed between the sampled adolescents and parents/guardians in the ethnolab phase, however. This was because the Ethnolab was run as FGDs with adolescents and caregivers separately to understand levers for behaviour change. This didn't require us to organise and analyse data by households.
- **Edits:** No changes made to the manuscript
- Line 220-223 talk about interview tool, structured around objectives and themes, but apparently these themes and objectives were not stated anywhere.
- **Rebuttal:** We have added text around how these themes were arrived at and some examples of the same as well.
- **Lines: 262 - 266**
- **Edits:** Some themes explored were, for example, anticipated loss of wages (while going through the procedure, while caring for the teen), anticipated conflict (when initiating conversation around EAMC). The themes used were identified from our previous research [Vaish et al., 2015], which were augmented, adapted and refined with findings from the formative research with adolescents and caregivers for the EAMC context.

*** These are observations and probes just for the immersion aspect of the method. Most of them, plus more, apply as well for the journey mapping and ethnolab phases

**Results and discussion**

Appreciable and commendable effort was made to present results with illustrative quotations. However:

- The large volumes of data and the absence of a clear purpose and objectives of the study makes the structure of this section difficult to follow.
- **Rebuttal:** We have added 2 tables to the manuscript in response to this point:

1. A table **(Line 224)** detailing out the time frame, sample and objectives of each phase of research to help improve understanding and readability of the three phases of research, and

2. A table **(Line 489)** detailing out how each phase of research sought to address the methodological challenges identified earlier in the paper

- **Edits:** Table 2 and Table 9
- The discussion is not well grounded in literature.
- **Rebuttal:** We have cited some supporting research across the results section
- **Lines:** **Multiple**
- **Edits:** Multiple

**Conclusion**

- In the absence of clear purpose and objectives from the start, this section becomes hanging.
- **Rebuttal:** We have re-worded the objectives **(Lines 73 – 84)** to improve the clarity on the scope and purpose of this paper.
- **Edits:** None
- Instead of describing the contributions of the different elements of the methodology, it would be more appropriate to give an overview of the key findings, and direct answers to the initial research questions.
- **Rebuttal:** Having clarified that the objective of the paper is to introduce a new methodological approach to studying joint decision making, we believe that the conclusions are now in line with the objectives that we set out to achieve. An overview of key takeaways has been included from **Lines 868 - 887** in the discussion section.
- **Edits:** None

**Other concerns**

- Participants were given incentives (lines 207 - 209), and that is often regarded as coercive and hence unethical.
- **Rebuttal:** While the respondents were not paid for participating in any of the research phases, they were compensated with a minimal amount for travel expenses and/or wage loss incurred due to the time allocated for research. This is what we have referred to as an 'incentive', which was merely used to offset any expenses participants may have incurred to participate in our research.
- **Edits:**

1. **Lines 247 – 249** - Participants were offered an amount of 100 Kwacha (~$5.00 USD) per household, and 50 Kwacha (~$2.50 USD) for school and facility staff, in lieu of any costs borne by them to participate in the study such as travel expenses, wage loss etc.

2. **Lines 389 - 391** - An amount of 100 Kwacha (~$5.00 USD) was paid to each participant to compensate them for their time or lost wages, if any.

- Language editing is also recommended for this submission
- **Rebuttal:** There are a few changes that we have made across the manuscript to improve ease of understanding and clarity -

1. Replaced 'son' with 'adolescent' and 'parent/guardian' with 'caregiver' across the document to make the language more consistent

2. Replaced 'immersion' with 'formative research' across the document to aid understanding of what this research phase entailed

3. Replaced 'VMMC for adolescents' with 'EAMC' to reduce the wordiness of the text

4. Replaced 'simulated decision-making environment' to 'simulated decision-making context' in the description of Ethnolab, as this describes our approach more accurately

- **Lines:** **Multiple**
- **Edits:** Multiple

**B.**	**OVERALL IMPRESSION AND RECOMMENDATION.**

- There seems to be too much content compressed into this manuscript, making it difficult to exhaust refined descriptions of each methodological aspect. Thus, there are some gaps in the methodological descriptions.
- **Rebuttal:** We have added 2 tables to the manuscript in response to this point: 1. A table **(Line 224)** detailing out the time frame, sample and objectives of each phase of research to help improve understanding and readability of the three phases of research, and 2. A table **(Line 489)** detailing out how each phase of research sought to address the methodological challenges identified earlier in the paper
- **Edits:** Table 2 and Table 9
- I would recommend that authors consider splitting the manuscript into 2 or 3 sizeable and refined manuscripts. For example, each of the 3 elements: the interviews, journey mapping and ethnolab can constitute a distinct manuscript, where all the refined details are included, along with rationales
- **Rebuttal:** The authors have made effort to streamline the problem statement, objectives and results section to make the connections clearer in an attempt to retain the refined details and rationales, while presenting our methodology more holistically.
- **Lines: Multiple**
- **Edits:** Multiple - This has been made across the manuscript based by incorporating the various points of feedback included by reviewers
